# Systematic review and meta-analysis of case-crossover and time-series studies of short term outdoor nitrogen dioxide exposure and ischemic heart disease morbidity

**DOI:** 10.1186/s12940-020-00601-1

**Published:** 2020-05-01

**Authors:** David M. Stieb, Carine Zheng, Dina Salama, Rania BerjawI, Monica Emode, Robyn Hocking, Ninon Lyrette, Carlyn Matz, Eric Lavigne, Hwashin H. Shin

**Affiliations:** 1 Environmental Health Science and Research Bureau, Health Canada, 420-757 West Hastings St. - Federal Tower, Vancouver, BC V6C 1A1 Canada; 2 School of Epidemiology and Public Health, University of Ottawa, Ottawa, Canada; 3 School of Population and Public Health, University of British Columbia, Vancouver, Canada; 4 Learning, Knowledge and Library Services, Health Canada, Ottawa, Canada; 5 Water and Air Quality Bureau, Health, Canada, Ottawa, Canada; 6 Department of Mathematics and Statistics, Queen’s University, Kingston, Canada

**Keywords:** Nitrogen dioxide, Ischemic heart disease, Time-series, Case-crossover

## Abstract

**Background:**

Nitrogen dioxide (NO_2_) is a pervasive urban pollutant originating primarily from vehicle emissions. Ischemic heart disease (IHD) is associated with a considerable public health burden worldwide, but whether NO_2_ exposure is causally related to IHD morbidity remains in question. Our objective was to determine whether short term exposure to outdoor NO_2_ is causally associated with IHD-related morbidity based on a synthesis of findings from case-crossover and time-series studies.

**Methods:**

MEDLINE, Embase, CENTRAL, Global Health and Toxline databases were searched using terms developed by a librarian. Screening, data extraction and risk of bias assessment were completed independently by two reviewers. Conflicts between reviewers were resolved through consensus and/or involvement of a third reviewer. Pooling of results across studies was conducted using random effects models, heterogeneity among included studies was assessed using Cochran’s Q and I^2^ measures, and sources of heterogeneity were evaluated using meta-regression. Sensitivity of pooled estimates to individual studies was examined using Leave One Out analysis and publication bias was evaluated using Funnel plots, Begg’s and Egger’s tests, and trim and fill.

**Results:**

Thirty-eight case-crossover studies and 48 time-series studies were included in our analysis. NO_2_ was significantly associated with IHD morbidity (pooled odds ratio from case-crossover studies: 1.074 95% CI 1.052–1.097; pooled relative risk from time-series studies: 1.022 95% CI 1.016–1.029 per 10 ppb). Pooled estimates for case-crossover studies from Europe and North America were significantly lower than for studies conducted elsewhere. The high degree of heterogeneity among studies was only partially accounted for in meta-regression. There was evidence of publication bias, particularly for case-crossover studies. For both case-crossover and time-series studies, pooled estimates based on multi-pollutant models were smaller than those from single pollutant models, and those based on older populations were larger than those based on younger populations, but these differences were not statistically significant.

**Conclusions:**

We concluded that there is a likely causal relationship between short term NO_2_ exposure and IHD-related morbidity, but important uncertainties remain, particularly related to the contribution of co-pollutants or other concomitant exposures, and the lack of supporting evidence from toxicological and controlled human studies.

## Background

Nitrogen dioxide (NO_2_) is a pervasive urban pollutant originating primarily from vehicle emissions, but also more broadly from any combustion in air [1, 2]. Other important contributors in areas with specific point sources include industrial sources and fossil fuel powered electric power generating stations [1, 2]. While ambient concentrations of NO_2_ have declined considerably in North America, Europe, Japan and South Korea, concentrations are increasing in other areas (e.g. China, North Korea and Taiwan) [3]. Numerous studies have evaluated health effects of nitrogen dioxide on diverse body systems. In particular, respiratory adverse effects have exhibited a relatively consistent association with NO_2_ in epidemiological studies, and these associations are supported by consistent toxicological and human clinical evidence of effects on the respiratory system [1, 2].

As a leading cause of morbidity and mortality worldwide, ischemic heart disease (IHD), including myocardial infarction and angina pectoris, is associated with a considerable public health burden [4]. Given its high prevalence, even relatively small incremental risks associated with air pollution exposure represent a substantial preventable burden on health. Nawrot et al. estimated that traffic exposure was associated with the largest population attributable fraction (PAF-7.4%) of all (including behavioural) triggers of myocardial infarction, while particulate matter was also associated with a substantial PAF (4.8%) [5]. However, whether NO_2_ exposure is causally related to IHD morbidity remains an unresolved question. A particular complicating factor is whether NO_2_ itself is to blame, or whether it is simply acting as a marker for specific air pollution sources i.e. emissions from vehicles [6, 7]. Carbon monoxide and certain chemical components of fine particulate matter, also primarily originating from vehicle emissions, are key potential confounders, given their well-established pathophysiological mechanisms of action on cardiac ischemia [8]. Effects of NO_2_ could also be confounded by other concomitant traffic-related exposures such as noise or stress [5]. We are aware of two previous systematic reviews/ meta-analyses which have evaluated the short term association of NO_2_ and IHD morbidity [9, 10]. These included primary studies published up to 2011 only, provided only limited evaluation of sources of heterogeneity, and did not examine whether the magnitude of effect differed between single and multi-pollutant models. In Mills et al.’s systematic review [10], study quality/risk of bias was not assessed. Only Mustafic et al. [9] and two other systematic reviews and meta-analyses have examined particulate matter and IHD morbidity [11, 12]. Our objective is therefore to determine whether short term exposure to outdoor NO_2_ is causally associated with morbidity from IHD based on an up to date synthesis of the available evidence.

## Methods

### Literature searches

MEDLINE, Embase, CENTRAL, Global Health and Toxline databases were searched using terms developed by a librarian (see Additional File 1). The search strategy underwent Peer Review of Electronic Search Strategies (PRESS) [13]. Searches were last updated August 27, 2019. Inclusion criteria were as follows: Participants/population: Humans; Intervention(s), exposure(s): Exposure to outdoor NO_2_ (and other oxides of nitrogen); Comparator(s)/control: Lower levels of exposure; Main outcomes: Counts of hospital admissions, emergency visits, physician office visits for IHD (including myocardial infarction (MI) and angina pectoris (AP)). Publications in abstract form only were excluded. Publications in English or French were included and there were no restrictions on publication date. Effect measures considered were: morbidity effects reported as regression coefficients, odds ratios or relative risks associated with exposures over days to weeks, expressed per specified increment in exposure. The present review is one part of a series of reviews of effects of NO_2_, all of which were included in the original search. Other reviews pertain to non-asthma respiratory morbidity related to short term exposure, and mortality related to long term exposure [14]. Studies were selected for the present review if reported outcomes matched the inclusion criteria specified above.

### Screening, data extraction and risk of bias assessment

Screening, data extraction and risk of bias assessment were completed independently by two reviewers in DistillerSR. Conflicts between reviewers were resolved through consensus and/or involvement of a third reviewer. All studies retrieved from literature searches were screened for relevance based on title and abstract according to the above inclusion criteria. Where relevance could not be determined based on abstract and title, the full text was reviewed. Manual searches were also completed of reference lists of all relevant studies. Bibliographic data, study location and timing, design, population age group(s), sample size, outcome (hospital admission, emergency visit, physician visit), diagnosis (including ICD code(s) if available), method of exposure assessment, pollutant (including name, averaging time, units, lag, descriptive statistics), type of regression model, effect measure and standard error or confidence interval, model covariates (potential confounders) and their specification were extracted from all studies meeting inclusion criteria. When single pollutant results were presented for multiple lag times, we extracted the most highly statistically significant result (regardless of the direction of the association), or that reported by the authors as their primary finding. Results from multi-pollutant models that resulted in the greatest reduction in magnitude of effect compared to single pollutant results were selected in order to bracket the magnitude of effect from each study. Results expressed per pollutant increment expressed in μg/m^3^ were converted to parts per billion [15], and those based on 1 h maximum exposures were multiplied by 1.9 (the average ratio of 1 h maximum to 24 h average NO_2_ in Canadian cities). Where required data were not provided, authors were contacted by e-mail. In some instances Engauge Digitizer [16] was employed to extract numeric results presented only in graph form. Modifications of the Navigation Guide systematic review methodology [17] based on earlier systematic reviews of time-series and case-crossover studies [9, 18–20] as well as methodological reviews [21, 22], were employed to evaluate risk of bias according to the following domains: exposure assessment, confounding, outcome assessment, completeness of outcome data, selective outcome reporting, conflict of interest and other sources of bias.

### Data analysis

The case-crossover approach can be regarded as an application of log-linear time series analysis if the time window of the case-crossover is comparable to the smoothing function on time in the time series [23]. However, since this condition may not be uniformly satisfied across all reviewed studies, and because case-crossover and time-series studies express effects using different measures of association (odds ratios and relative risks respectively), we analyzed them separately. Pooling of results across studies was conducted using random effects models computed using Restricted Maximum Likelihood (REML) estimation, with sensitivity analyses employing Dersimonian and Laird and Empirical Bayes estimators [24]. Heterogeneity among included studies was assessed using Cochran’s Q and I^2^ measures, and sources of heterogeneity were evaluated using meta-regression [24]. Sensitivity of pooled estimates to individual studies was examined using Leave One Out analysis and publication bias was evaluated using Funnel plots, Begg’s and Egger’s tests, and trim and fill [24]. Subgroup analyses were conducted by region, age group, sex, and single vs. multi-pollutant models. Analysis was conducted in R version 3.6.0 [25] using the metafor package [24]. The systematic review protocol is registered with PROSPERO (CRD42018084497) [14].

## Results

**Fig. 1:**
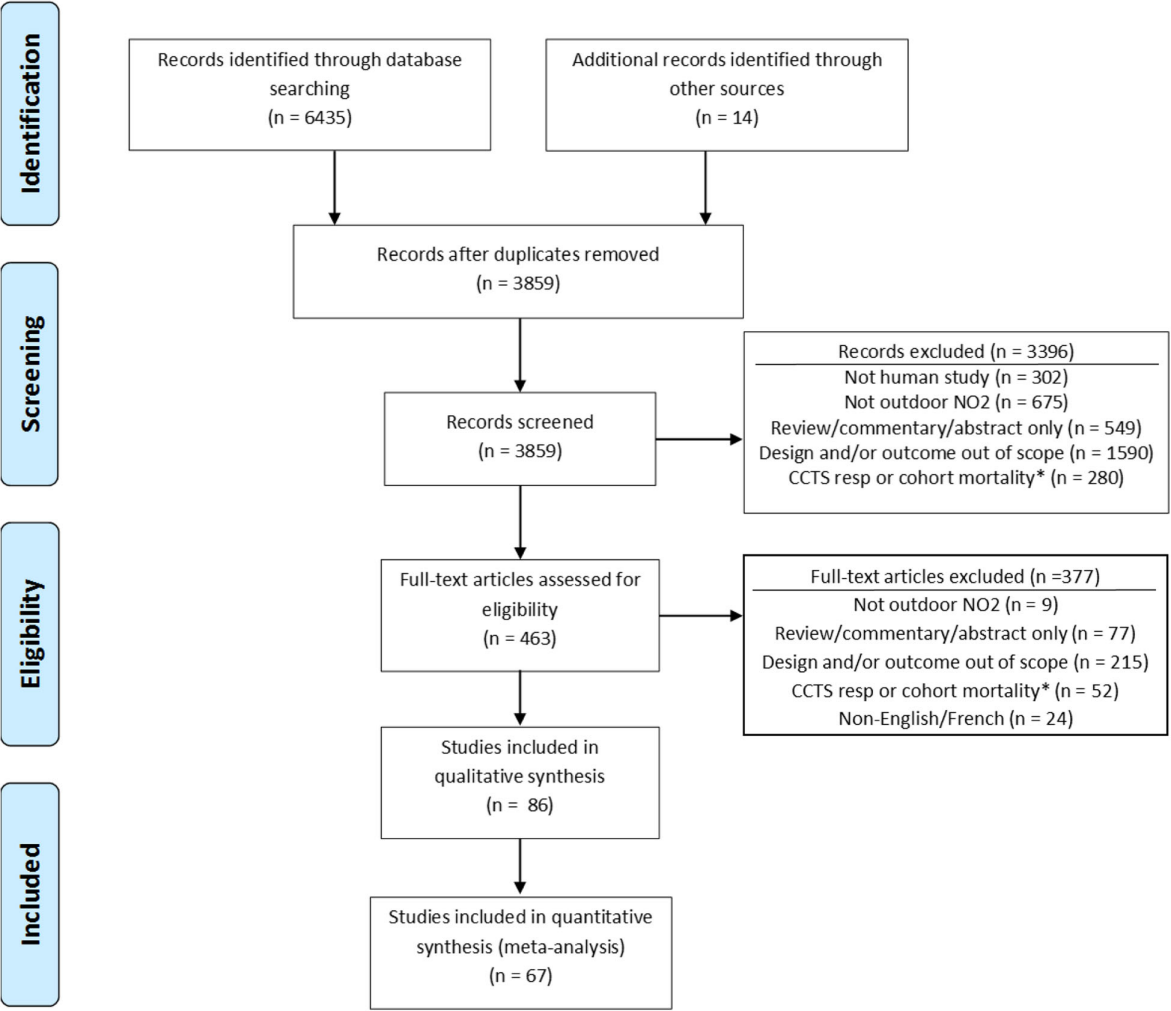
Preferred Reporting Items for Systematic Reviews and Meta-Analyses (PRISMA) flow diagram

A Preferred Reporting Items for Systematic Reviews and Meta-Analyses (PRISMA) diagram summarizing disposition of studies identified in literature searches is shown in Fig. 1. As indicated earlier, the present review is one part of a series of reviews of effects of NO_2_ on multiple outcomes, all of which were included in the original search, which is reflected in numeric results reported in Fig. 1. Thirty-eight case-crossover studies [26–63] and 48 time-series studies [64–111] were included in our final analysis. Study characteristics are summarized in Tables 1 and 2. The majority of case-crossover studies, *n* = 27 (71%), and time-series studies, *n* = 26 (54%), were conducted in Europe or North America and most, *n* = 62 of 86 total (72%), were based on single cities. Almost all studies, *n* = 84 (98%), employed monitoring (vs. modelling) as the source of exposure data, and most, *n* = 70 (81%), employed 24 h average concentration as the exposure metric. Most studies, *n* = 72 (84%), were based in whole or in part on hospital admission data. MI was the most commonly evaluated outcome, *n* = 55 studies (64%), and 14 studies (16%) examined subtypes (ST-elevation or transmural vs. Non-ST elevation). Thirty seven studies (43%) were mostly conducted prior to 2000 (majority of study duration prior to 2000) while 49 (57%) were conducted mostly post 2000. In total, analyses in the included studies were based on over 3.2 million events (the actual total is larger, but not all studies reported the number of events), and the number of events in individual studies ranged from 53 to 630,116.

**Table 1:** Summary of case-crossover study characteristics

Study	Country/Region	Location	Start	End	Events	Outcome^a^	Diagnosis^b^	Exposure	Mean NO_2_ (ppb)^c^
Wang 2015 [26]	Canada	Calgary, Edmonton, Canada	1999	2010	22,628	HA	AMI, NSTEMI, STEMI	Monitor	NA
Wang 2015 [27]	Canada	Alberta, Canada	1999	2010	25,894	HA	AMI	Monitor	15.0
Weichenthal 2016 [28]	Canada	Ontario, Canada	2004	2011	30,101	EV	AMI	Monitor	12.3
Weichenthal 2016 [29]	Canada	Ontario, Canada	2004	2011	17,960	EV	AMI	Monitor	14.1
Basu 2012 [30]	United States	California, US	2005	2008	32,890	EV	IHD	Monitor	14.9
Evans 2017 [31]	United States	Rochester, US	2007	2012	366	HA	STEMI	Monitor	4.3
Peel 2007 [32]	United States	Atlanta, US	1993	2000	32,731	EV	IHD	Monitor	24.2
Peters 2001 [33]	United States	Boston, US	1995	1996	772	HA	AMI	Monitor	24.0
Rich 2010 [34]	United States	New Jersey, US	2004	2006	1262	HA	STEMI	Monitor	NA
Zanobetti 2006 [35]	United States	Boston, US	1995	1999	15,578	HA	AMI	Monitor	13.6
Argacha 2016 [36]	Europe	Belgium	2009	2013	11,428	HA	STEMI	Monitor	12.6
Bard 2014 [37]	Europe	Strasbourg, France	2000	2007	2134	HA	AMI	Model	17.8
Berglind 2010 [38]	Europe	Stockholm, Sweden	1993	1994	660	HA	AMI	Monitor	13.8
Bhaskaran 2011 [39]	Europe	England, Wales	2003	2006	79,288	HA	AMI	Monitor	8.7
Buszman 2018 [40]	Europe	3 Polish cities	2014	2015	1957	HA	NSTEMI,STEMI	Monitor	9.5
Butland 2016 [41]	Europe	England, Wales	2003	2010	630,116	HA	AMI, NSTEMI, STEMI	Monitor	9.0
Collart 2015 [42]	Europe	Charleroi, Belgium	1999	2008	2859	HA	AMI	Monitor	18.7
D’Ippoliti 2003 [43]	Europe	Rome, Italy	1995	1997	6531	HA	AMI	Monitor	45.9
Milojevic 2014 [44]	Europe	England and Wales	2003	2009	452,343	HA	AMI, NSTEMI, STEMI	Monitor	13.8
Nuvolone 2011 [45]	Europe	Tuscany, Italy	2002	2005	11,450	HA	AMI	Monitor	NA
Panasevich 2013 [46]	Europe	Stockholm, Sweden	1992	1994	1192	HA	AMI	Monitor	13.7
Peters 2005 [47]	Europe	Augsburg, Germany	1999	2001	851	HA	AMI	Monitor	19.0
Ruidavets 2005 [48]	Europe	Toulouse, France	1997	1999	399	HA	AMI	Monitor	16.7
Sahlen 2019 [49]	Europe	Stockholm, Sweden	2000	2014	14,601	HA	STEMI	Monitor	8.0
Vencloviene 2011 [50]	Europe	Kaunas City, Lithuania	2004	2006	6594	HA	AMI	Monitor	18.4
Wichmann 2012 [51]	Europe	Copenhagen, Denmark	1999	2006	14,456	HA	AMI	Monitor	12.0
Wichmann 2013 [52]	Europe	Gothenburg, Sweden	1985	2010	24,355	HA	AMI	Monitor	14.5
Akbarzadeh 2018 [53]	Other	Tehran, Iran	2014	2016	208	HA	STEMI	Monitor	60.7
Barnett 2006 [54]	Other	7 cities in New Zealand, Australia	1998	2001	NA	HA	AMI	Monitor	9.2
Cheng 2009 [55]	Other	Kaohsiung, Taiwan	1996	2006	9349	HA	AMI	Monitor	26.5
Franck 2014 [56]	Other	Santiago, Chile	2004	2007	15,296	HA	IHD	Monitor	18.1
Hsieh 2010 [57]	Other	Taipei, Taiwan	1996	2006	23,420	HA	AMI	Monitor	29.9
Huang 2016 [58]	Other	Taiwan	2000	2013	1835	EV&HA	IHD	Monitor	NA
Kojima 2014 [59]	Other	Kumamoto, Japan	2010	2015	3713	HA	AMI	Monitor	10.5
Li 2019 [60]	Other	Yancheng, China	2015	2018	347	HA	STEMI	Monitor	10.9
Liu 2017 [61]	Other	China	2014	2015	80,787	HA	AMI	Monitor	24.8
Tsai 2012 [62]	Other	Taipei, Taiwan	1999	2009	27,563	HA	AMI, IHD	Monitor	27.6
Turin 2012 [63]	Other	Takashima, Japan	1988	2004	429	HA, other	AMI	Monitor	16.0

^a^*HA* Hospital admission; *EV* Emergency visit; ^b^*AMI* Acute myocardial infarction; *IHD* Ischemic heart disease; *NSTEMI* Non ST-elevation MI; *STEMI* ST-elevation MI; ^c^24 hour average; in some cases estimated from median and/or daily 1 h maximum

**Table 2:** Summary of time-series study characteristics

Study	Country/Region	Location	Start	End	Events	Outcome^a^	Diagnosis^b^	Exposure	Mean NO_2_ (ppb)^c^
Burnett 1999 [64]	Canada	Toronto, Canada	1980	1994	131,496	HA	IHD	Monitor	25.2
Stieb 2000 [65]	Canada	Saint John, Canada	1992	1996	2435	EV	IHD	Monitor	8.9
Stieb 2009 [66]	Canada	7 Canadian cities	1992	2003	63,184	EV	IHD	Monitor	18.3
Szyszkowicz 2007 [67]	Canada	Montreal, Canada	1997	2002	4979	EV	IHD	Monitor	19.4
Krall 2018 [68]	United States	5 U.S. cities	2002	2008	NA	EV	IHD	Model	10.8
Linn 2000 [69]	United States	Los Angeles, US	1992	1995	NA	HA	AMI	Monitor	34.3
Lippmann 2000 [70]	United States	Detroit, US	1992	1994	NA	HA	IHD	Monitor	21.3
Mann 2002 [71]	United States	Southern California, US	1988	1995	19,690	HA	AMI	Monitor	37.2
Metzger 2004 [72]	United States	Atlanta, US	1993	2000	32,762	EV	IHD	Monitor	24.2
Pearce 2018 [73]	United States	Columbia, US	2002	2013	307,313	HA	IHD	Monitor	7.8
Sarnat 2015 [74]	United States	St. Louis, US	2001	2003	22,097	EV	IHD	Monitor	16.5
Anderson 2001 [75]	Europe	West Midland, UK	1994	1996	NA	HA	IHD	Monitor	19.6
Atkinson 1999 [76]	Europe	London, UK	1992	1994	NA	HA	IHD	Monitor	50.3
Baneras 2018 [77]	Europe	Barcelona, Spain	2010	2011	4141	HA	STEMI	Monitor	18.7
Caussin 2015 [78]	Europe	Paris, France	2003	2008	11,987	HA	STEMI	Monitor	20.8
Collart 2018 [79]	Europe	Wallonia, Belgium	2008	2011	21,491	HA	AMI	Monitor	10.9
Eilstein 2001 [80]	Europe	Strasbourg, France	1984	1989	1491	HA, other	AMI	Monitor	28.9
Halonen 2009 [81]	Europe	Helsinki, Finland	1998	2004	NA	HA	IHD	Monitor	16.0
Konduracka 2019 [82]	Europe	Krakow, Poland	2012	2015	3545	HA	AMI	Monitor	29.2
Lanki 2006 [83]	Europe	5 European cities	1992	2000	26,854	HA	AMI	Monitor	NA
Larrieu 2007 [84]	Europe	8 French cities	1998	2003	NA	HA	IHD	Monitor	17.6
Le Tertre 2002 [85]	Europe	8 European cities	1989	1997	NA	HA	IHD	Monitor	30.5
Medina 1997 [86]	Europe	Paris, France	1991	1995	NA	MD	IHD	Monitor	29.8
Poloniecki 1997 [87]	Europe	London, UK	1987	1994	67,448	HA	AMI	Monitor	36.2
Ponka 1996 [88]	Europe	Helsinki, Finland	1987	1989	12,664	HA	IHD	Monitor	20.7
von Klot 2005 [89]	Europe	5 European cities	1992	2000	2321	HA	AMI	Monitor	26.4
Bell 2008 [90]	Other	Taipei, Taiwan	1995	2002	6909	HA	IHD	Monitor	26.4
Cendon 2006 [91]	Other	Sao Paolo, Brazil	1998	1999	19,058	HA	AMI	Monitor	28.0
Chen 2019 [92]	Other	Jinan, China	2013	2015	11,583	HA	AMI	Monitor	30.3
Ghaffari 2017 [93]	Other	Tabriz, Iran	2011	2013	NA	HA	STEMI	Monitor	NA
Goggins 2013 [94]	Other	Hong Kong & Kaohsiung, Taipei, Taiwan	2000	2009	84,328	HA	AMI	Monitor	30.1
Hosseinpoor 2005 [95]	Other	Tehran, Iran	1996	2001	42,880	HA	AP	Monitor	31.9
Jalaludin 2006 [96]	Other	Sydney, Australia	1997	2001	28,855	EV	IHD	Monitor	12.2
Lee 2003 [97]	Other	Seoul, Korea	1997	1999	10,193	HA	AP, IHD	Monitor	31.5
Phosri 2019 [98]	Other	Bangkok, Thailand	2006	2014	26,298	HA	AMI	Monitor	22.2
Pothirat 2019 [99]	Other	Chiang Mai, Thailand	2016	2017	53	EV&HA	AMI	Monitor	15.9
Qiu 2013 [100]	Other	Hong Kong	1998	2007	110,123	HA	IHD	Monitor	30.8
Simpson 2005 [101]	Other	4 Australian cities	1996	1999	126,377	HA	IHD	Monitor	11.2
Soleimani 2019 [102]	Other	Shiraz, Iran	2009	2015	6425	HA	AMI	Monitor	19.0
Tam 2015 [103]	Other	Hong Kong	2001	2010	NA	HA	IHD	Monitor	30.3
Thach 2010 [104]	Other	Hong Kong	1996	2002	117,866	HA	IHD	Monitor	31.2
Wong 1999 [105]	Other	Hong Kong	1994	1995	NA	HA	IHD	Monitor	28.5
Wong 2002 [106]	Other	Hong Kong, London, UK	1992	1997	95,681	HA	IHD	Monitor	29.7
Xie 2014 [107]	Other	Shanghai, China	2010	2012	47,523	EV	AMI	Monitor	29.8
Yamaji 2017 [108]	Other	Japan	2011	2012	56,863	HA	STEMI	Monitor	14.2
Ye 2001 [109]	Other	Tokyo, Japan	1980	1995	NA	EV	AMI	Monitor	25.4
Yu 2013 [110]	Other	Hong Kong	1998	2007	109,983	HA	IHD	Monitor	30.8
Yu 2018 [111]	Other	Changzhou, China	2015	2016	5545	HA	AMI	Monitor	20.7

^a^HA, hospital admission; EV, emergency visit, MD, physician visit; ^b^AMI, acute myocardial infarction; AP, Angina Pectoris; IHD, ischemic heart disease; NSTEMI, non ST-elevation MI; STEMI, ST-elevation MI; ^c^24 hour average; in some cases estimated from median and/or daily 1 h maximum

**Fig. 2:**
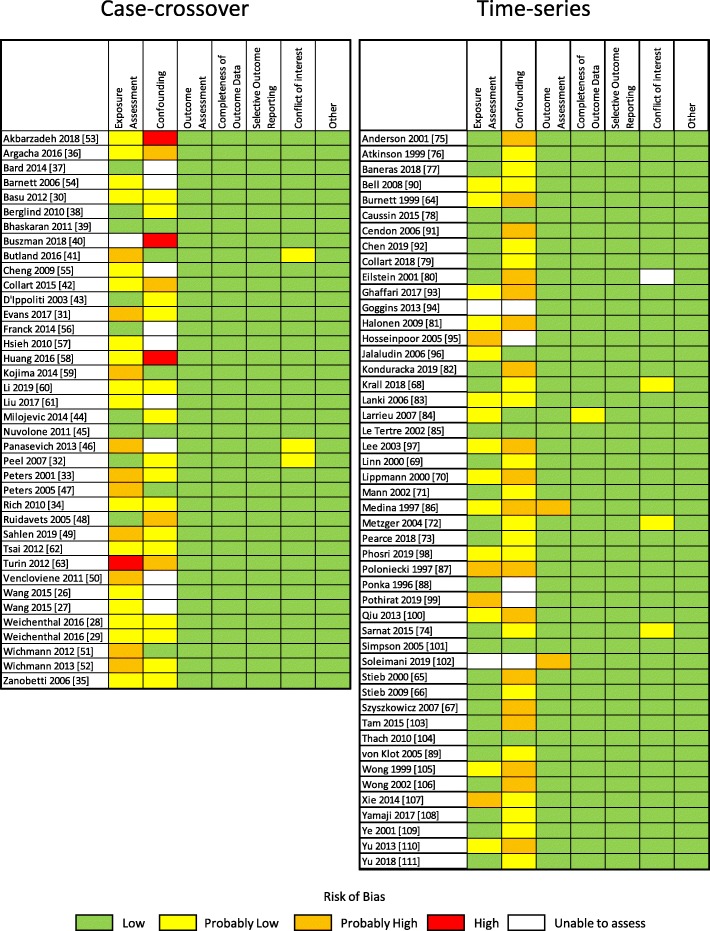
Summary of risk of bias ratings

Risk of bias ratings are summarized in Fig. 2, criteria are detailed in Additional File 2, and reasons for assigned ratings of risk of bias greater than low risk (or unable to assess) for individual studies are provided in Additional File 3. The greatest variability in ratings occurred in the exposure assessment and confounding domains, while ratings in the other domains (outcome assessment, completeness of outcome data, selective outcome reporting, conflict of interest, other sources of bias) were generally low or probably low risk of bias. Eighteen studies (20.9%) were rated probably high or high risk of bias or unable to assess in the exposure assessment domain because they relied on a single monitor, there was evidence of a mediocre correlation of modelled or measured values with ground measurements in the target community, or there was insufficient information. Forty studies (46.5%) were rated probably high or high risk of bias or unable to assess in the confounding domain because of lack of justification for covariate specification, employment of non-parametric smoothing functions associated with known biases [112, 113], unidirectional referent selection in case-crossover studies [22], or failure to describe covariate specification.

### Effect estimates and pooled effect estimates

**Fig. 3:**
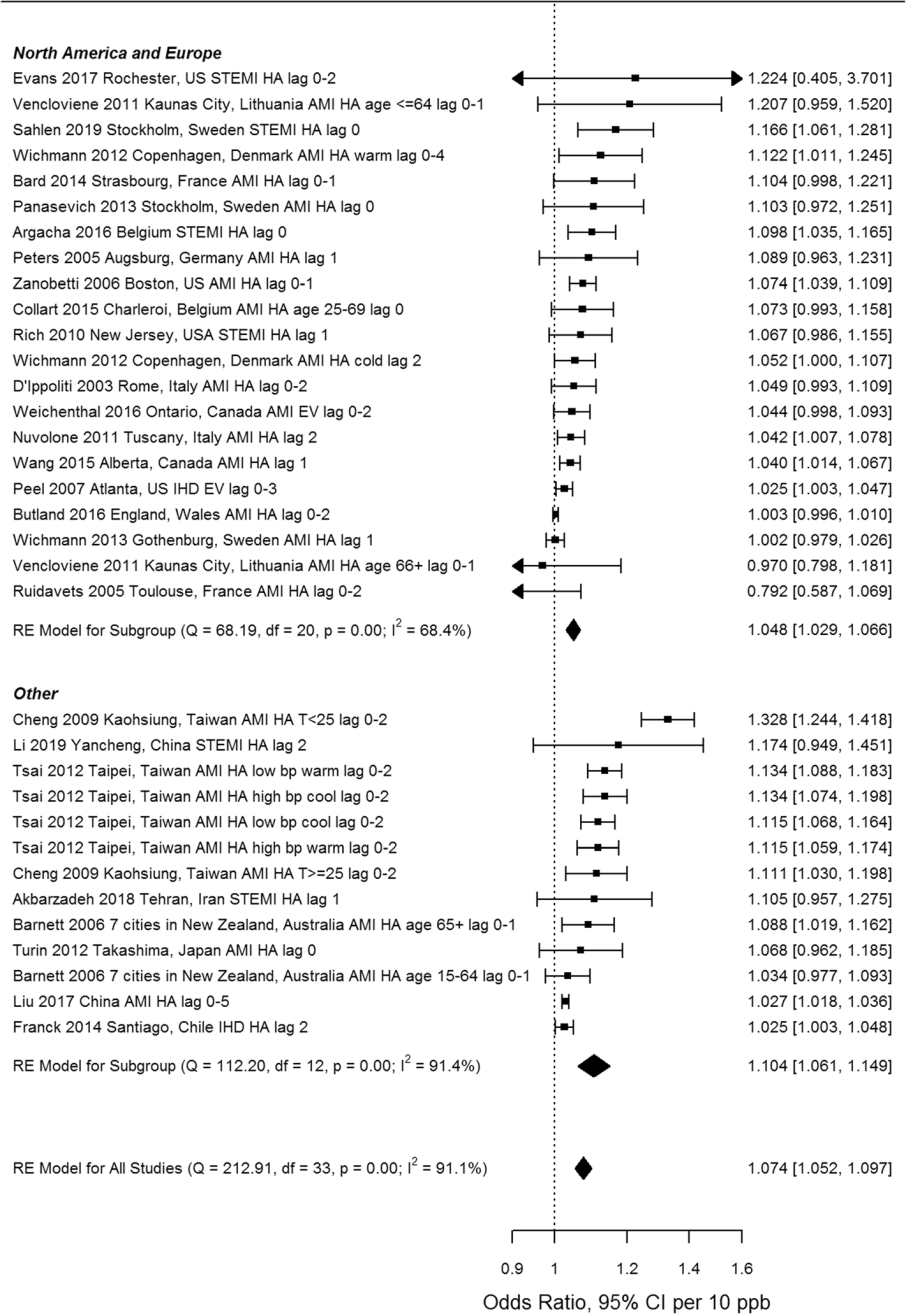
Odds ratios from single pollutant models from individual case-crossover studies and pooled estimates by region (AMI, acute myocardial infarction; IHD, ischemic heart disease; STEMI, ST-elevation MI; EV, emergency visit; HA, hospital admission; T, temperature; lag reported in days)

**Fig. 4:**
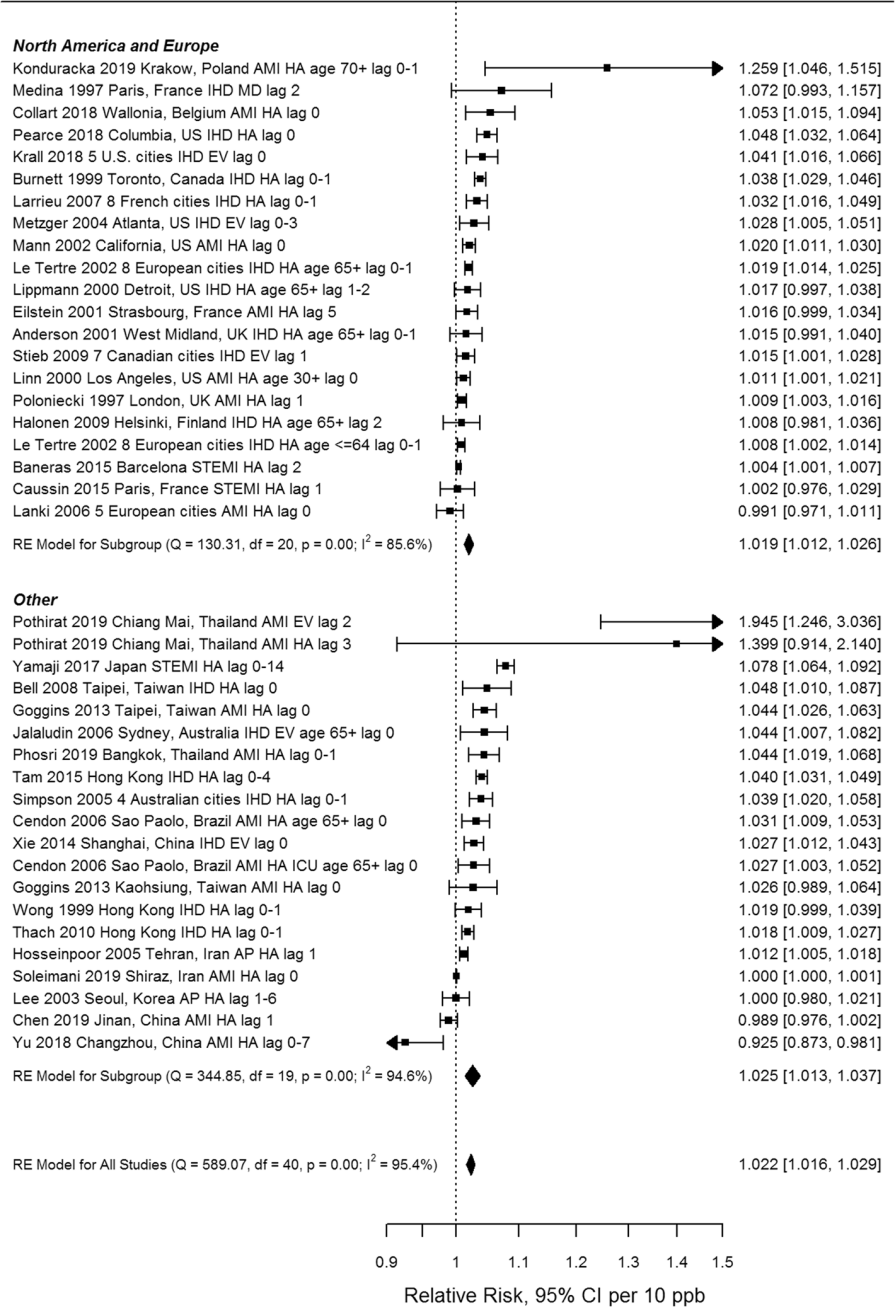
Relative risks from single pollutant models from individual time-series studies and pooled estimates by region (AMI, acute myocardial infarction, AP, angina pectoris, IHD, ischemic heart disease, STEMI, ST-elevation MI, EV, emergency visit, HA, hospital admission, MD, physician visit, lag reported in days)

**Fig. 5:**
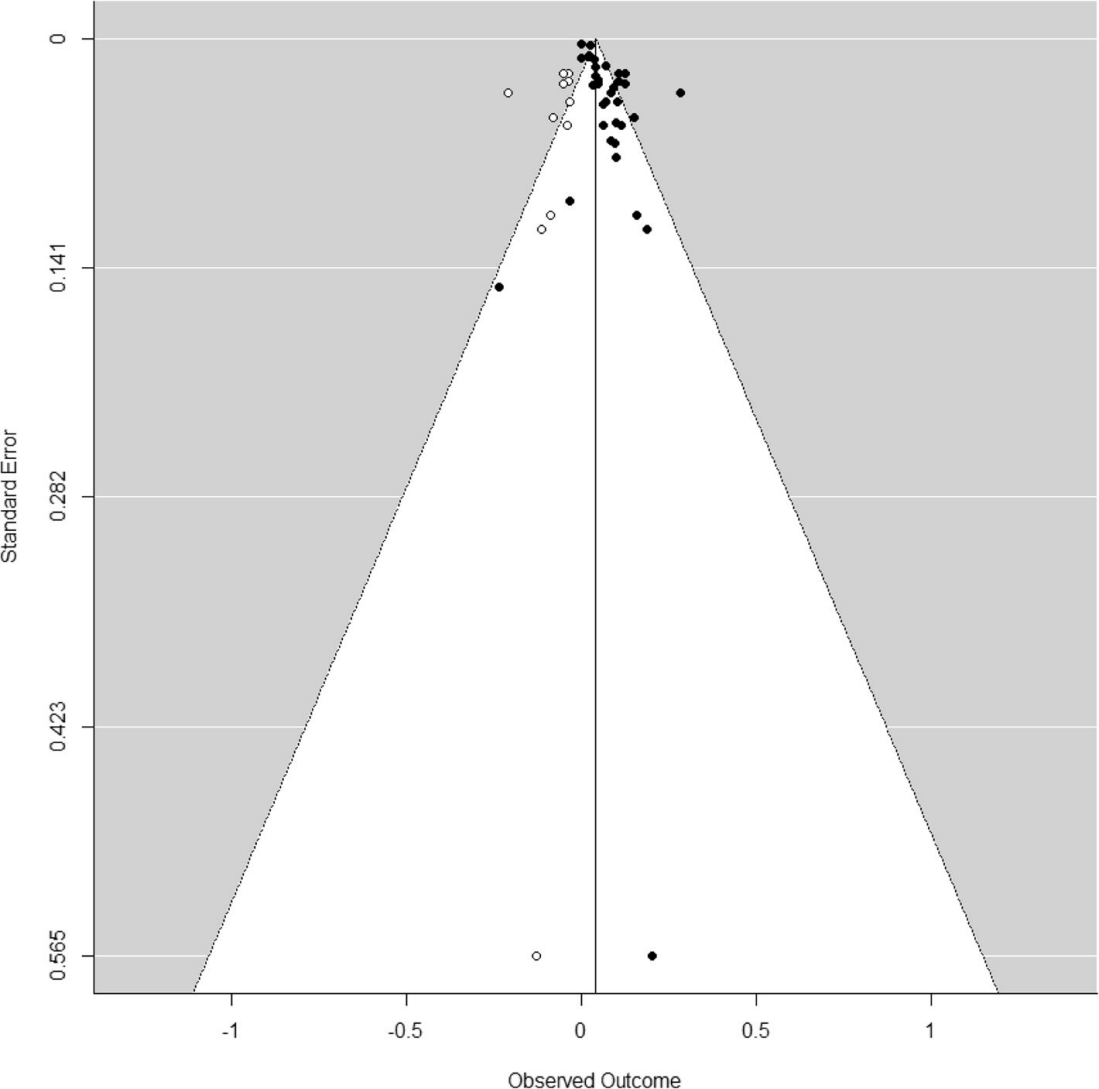
Funnel plot of log (Odds Ratio) vs. standard error for case-crossover studies from Fig. 3. Filled circles represent observed values, open circles represent missing studies identified with trim and fill, and the vertical line represents the log of the pooled odds ratio. In the absence of publication bias, points should be symmetrically distributed around the vertical line, with smaller studies (larger standard errors on vertical axis) more widely scattered. Filling the plot with points mirroring observed values corrects for apparently missing smaller and/or negative studies which may have been suppressed due to publication bias

All 189 extracted risk estimates from individual studies, including from single and multi-pollutant models, and by population and outcome subgroup are provided in forest plots by region in Additional Files 4-7. Of these, we excluded estimates from pooling if they pertained to a single season, were superseded by other studies encompassing the same geographic area or time frame e.g. in subsequent multi-city studies or those spanning a longer study duration, leaving 67 studies(28 case-crossover and 39 time-series) included in the meta-analysis. Forest plots of odds ratios and 95% confidence intervals based on single pollutant models from case-crossover studies, by region and overall, are shown in Fig. 3. Ninety-five percent confidence intervals on pooled estimates by region and overall excluded 1 or no effect (i.e. they were statistically significant). The pooled estimate for European and North American studies was lower than that for studies from other areas, and the difference was statistically significant (*p* = 0.019) (see Table 3). Heterogeneity was lower for European and North American studies (I^2^ = 68.4%) than for studies from other regions (I^2^ = 91.4%). Forest plots of relative risks and 95% confidence intervals based on single pollutant models from time-series studies, by region and overall, are shown in Fig. 4. Again, 95% confidence intervals on pooled estimates by region and overall excluded 1 or no effect, although the magnitude of effects was smaller than for case-crossover studies. Heterogeneity was uniformly high. The pooled estimate for European and North American studies was lower than that for studies from other areas, but the difference was not statistically significant (*p* = 0.40) (see Table 3). Pooled estimates were not sensitive to pooling estimator (REML vs. Dersimonian and Laird vs. Empirical Bayes) (Additional File 8), or to individual studies based on Leave One Out analysis (Additional File 9). Begg’s test of funnel plot asymmetry was not significant for either case-crossover or time-series studies, while Egger’s test indicated significant asymmetry for time-series studies (*p* = 0.002). Application of trim and fill (employing the L_0_ estimator [114]) to case-crossover studies was indicative of publication bias, suggesting that there were 11 missing studies with effect estimates less than the pooled estimate (Fig. 5). Filling in these studies was estimated to substantially reduce the overall pooled estimate for case-crossover studies from 1.074 (95%CI 1.052–1.097) to 1.044 (95% CI 1.017–1.070) per 10 ppb. Similarly, application of trim and fill to time-series studies suggested that there were 7 missing studies with effect estimates less than the pooled estimate. Filling in these studies was estimated to slightly reduce the overall pooled estimate for time-series studies from 1.022 (95%CI 1.016–1.029) to 1.019 (95%CI 1.012–1.026) per 10 ppb. See Additional File 10 for Funnel plot of time-series studies.

**Table 3:** Summary of subgroup analyses

Subgroup	Analysis	n	OR/RR	L95%CI	U95%CI	Q	p(Q)	I^2^ (%)	p (difference)
Case-crossover									
None	All single pollutant	34	1.074	1.052	1.097	212.91	< 0.01	91.1	
Region	North America, Europe	21	1.048	1.029	1.066	68.19	< 0.01	68.4	
	Other	13	1.104	1.061	1.149	112.20	< 0.01	91.4	0.019
Single/Multi pollutant	Single pollutant	9	1.075	1.019	1.135	95.89	< 0.01	98.2	
	Multi-pollutant	9	1.038	0.995	1.083	41.65	< 0.01	96.2	0.23
Sex	female	8	1.050	1.004	1.098	27.93	< 0.01	64.8	
	male	8	1.032	1.006	1.058	14.66	0.04	52.0	0.51
Age	younger	8	1.023	0.997	1.05	14.25	0.05	43.3	
	older	10	1.044	1.02	1.07	16.52	0.06	43.1	0.26
Time-series									
None	All single pollutant	41	1.022	1.016	1.029	589.07	< 0.01	95.4	
Region	North America, Europe	21	1.019	1.012	1.026	130.31	< 0.01	85.6	
	Other	20	1.025	1.013	1.037	344.85	< 0.01	94.6	0.40
Single/Multi Pollutant	Single pollutant	8	1.013	1.003	1.023	86.79	< 0.01	94.3	
	Multi-pollutant	9	1.008	0.998	1.018	34.14	< 0.01	81.0	0.49
Age	younger	7	1.015	1.001	1.029	37.78	< 0.01	92.1	
	older	8	1.033	1.011	1.056	65.01	< 0.01	95.2	0.18

### Meta-regression

Meta-regression revealed that the magnitude of the log odds ratio from case-crossover studies was significantly positively associated with study mean NO_2_ exposure (*p* = 0.042), as well as region other than North America or Europe (*p* = 0.033; there was no significant difference between North America and Europe), and timing of study primarily post 2000 (*p* = 0.031). When considered jointly, only region remained a nearly significant predictor (*p* = 0.057). Log relative risks from time-series studies were negatively associated with study mean NO_2_ (*p* = 0.041). Risk of bias in the exposure assessment and confounding domains, outcome (hospital admission vs. other), diagnosis (MI vs other), study interquartile range, standard deviation and range of NO_2_ were not significant predictors of the magnitude of effect for either case-crossover or time-series studies. Residual heterogeneity remained relatively high (I^2^ generally > 70%) even after accounting for significant predictor variables for both case-crossover and time-series studies.

### Single vs. multi-pollutant models and subgroup analyses

**Fig. 6:**
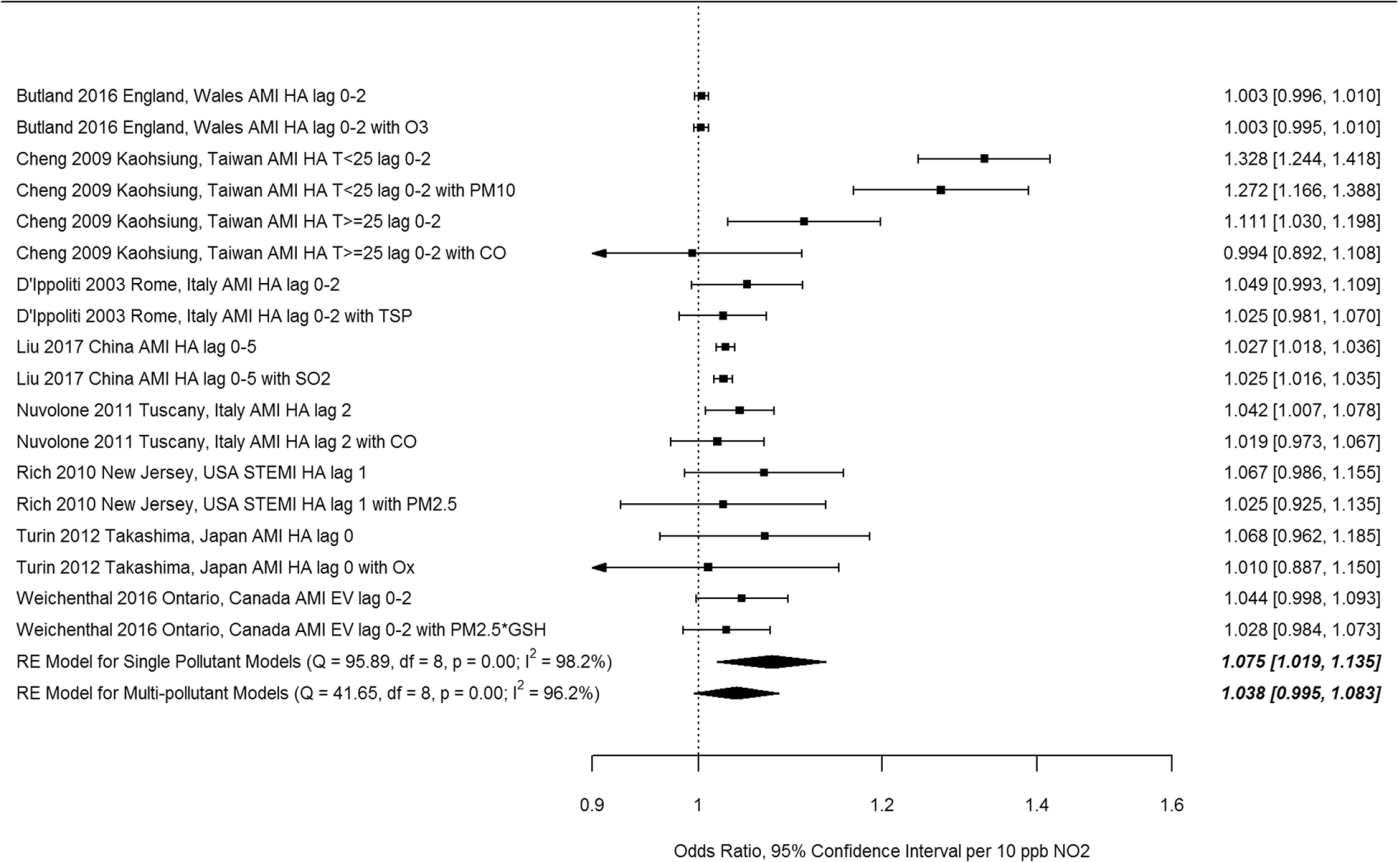
Odds ratios from individual case-crossover studies and pooled estimates from single and multi-pollutant models (AMI, acute myocardial infarction, STEMI, ST-elevation MI, EV, emergency visit, HA, hospital admission, T, temperature, Ox, total oxidants, GSH, glutathione related oxidative potential)

**Fig. 7:**
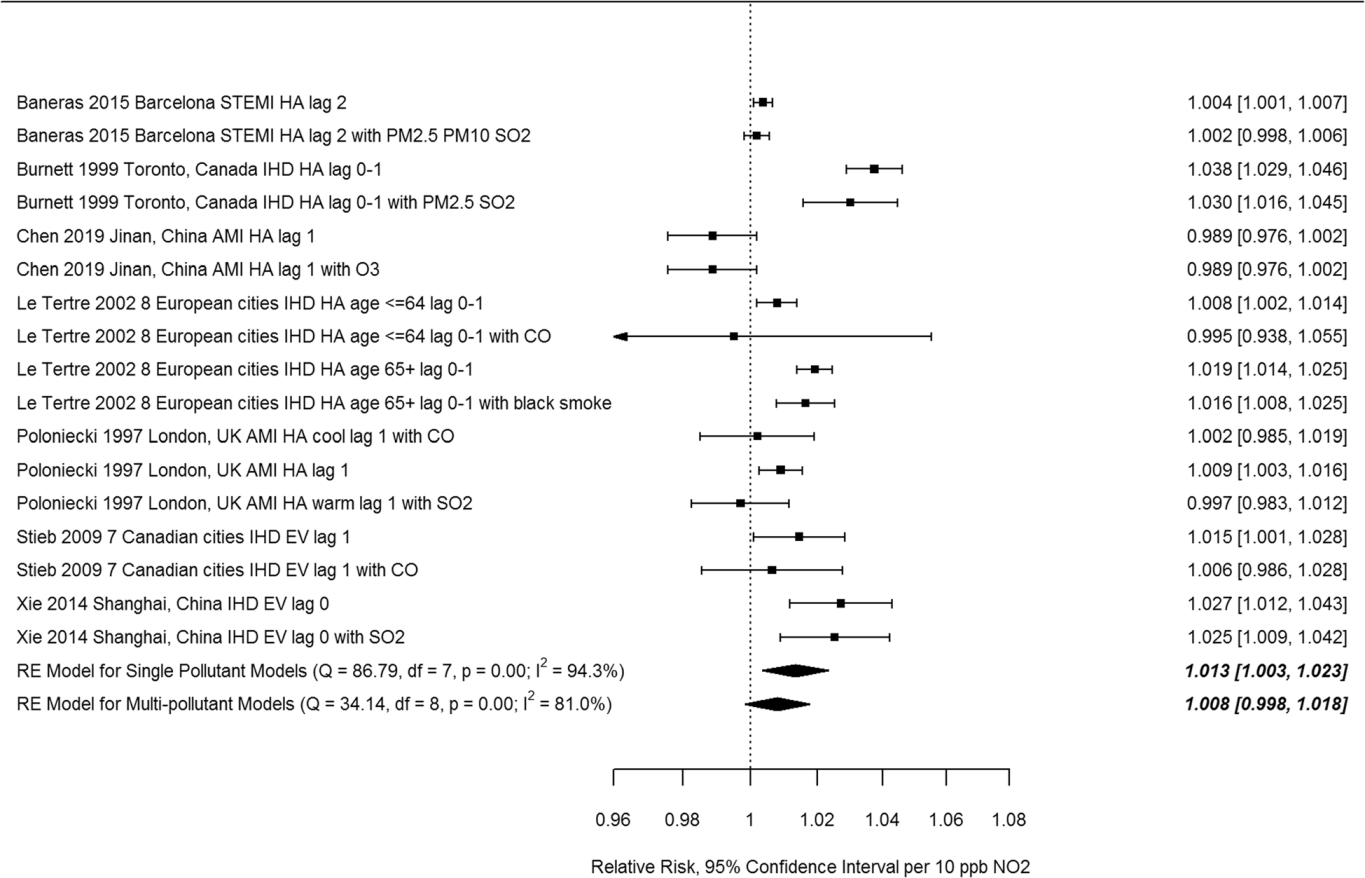
Relative risks from individual time series studies and pooled estimates from single and multi-pollutant models (AMI, acute myocardial infarction, IHD, ischemic heart disease, EV, emergency visit, HA, hospital admission)

Forest plots of paired estimates of effects from single and multi-pollutant models from the same study are shown in Figs. 6 and 7. Pooled estimates from single pollutant models were higher than those from multi-pollutant models and the confidence interval for multi-pollutant pooled estimates overlapped 1 or no effect. However, the difference between pooled estimates for single and multi-pollutant models was not significant (see Table 3).

Subgroup analyses are summarized in Table 3 in comparison to primary results. Pooled effect estimates were larger in older populations (generally ≥65 years, but in some cases ≥55 years or ≥ 75 years) in contrast to pooled estimates for younger populations for both case-crossover and time-series studies. However, differences between pooled estimates were not significant. No significant differences were observed by sex.

### Shape of exposure-response relationship

Thirteen studies evaluated the shape of the exposure-response relationship between NO_2_ and IHD morbidity by examining the association by quantile of NO_2_ [43, 48, 50, 110], plotting the association using a non-linear function of NO_2_ [76, 80, 94, 98, 103, 106, 107, 111], or testing the significance of the difference between linear and non-linear models [41]. Of these, eight studies found a linear association [41, 43, 50, 80, 94, 98, 106, 107], in some instances only in subsets of the data by age [50] or season [80], while three found evidence of a threshold [76, 103, 110], although the available evidence is insufficient to identify a precise threshold value. Two studies reported no association between NO_2_ and MI risk, based on analysis by quantiles [48], and a plot using a non-linear function of NO_2_ [111]. An additional case-crossover study not included in pooled estimates because it characterized exposure using fixed increment/decrement thresholds rather than a linear term, found an apparently linear association between rapid changes in NO_2_ concentration and odds of MI [115].

## Discussion

Based on an analysis of 67 case-crossover and time-series studies, we found that short term exposure to NO_2_ was significantly associated with IHD morbidity (pooled OR from case-crossover studies: 1.074 95% CI 1.052–1.097; pooled RR from time-series studies: 1.022 95% CI 1.016–1.029 per 10 ppb). There was evidence of publication bias particularly for case-crossover studies. Pooled estimates based on both types of studies were characterized by a high degree of heterogeneity. For case crossover studies, heterogeneity was only partially accounted for by study region (larger magnitude of effect outside Europe and North America), mean exposure (larger magnitude of effect at higher mean exposure), and age of study (larger magnitude of effect in newer studies), although when these factors were considered jointly, only study region was associated with magnitude of effect. Similarly, for time-series studies, heterogeneity was only partially accounted for by study mean NO_2_ (lower magnitude of effect with increasing mean). While risk of bias due to exposure assessment and confounding were not associated with magnitude of effect, residual heterogeneity could nonetheless be attributable to these factors, since we had only categorical ratings rather than precisely quantified measures of these factors. It is well documented, for example, that exposure measurement error is related to observed magnitude of effect, depending on type of error (classical or Berkson’s) [116–118]. Case-crossover and time-series studies are not confounded by risk factors related to individual characteristics which are stable over short time periods, as these are controlled for by design. Confounding by time is controlled for by design in case-crossover studies and by analysis in time-series studies, while confounding by time-varying factors such as weather, other pollutants and influenza epidemics is adjusted for in the analysis in both types of studies. We accounted for these factors through our assessment of risk of bias, and consideration of results from single and multi-pollutant models. We could not account for residual confounding by concomitant exposures to noise or stress which could be associated with both NO_2_ exposure and triggering of IHD morbidity, as these were not assessed in the primary studies we evaluated. Peters et al. [47, 119] collected data on time spent in traffic prior to MI onset and found that it was significantly associated with MI, but did not report joint models including both this variable and NO_2_ exposure. Pooled estimates based on multi-pollutant models were smaller than those from single pollutant models for both case-crossover and time-series studies, although these differences were not statistically significant. Pooled estimates based on older populations were also larger than those based on younger populations for both case-crossover and time-series studies, but again these differences were not statistically significant.

Our results are generally consistent with those of Mustafic et al., who included 21 studies in their meta-analysis and reported a pooled estimate of 1.011 (95% CI 1.006–1.016) per 10 μg/m^3^ NO_2_, with an I^2^ of 71% [9]. This is comparable to our pooled estimate for time-series studies (after converting to ppb), but smaller than that for case-crossover studies. Owing to the smaller number of studies, they were not able to evaluate results from single and multi-pollutant models, or for subgroups based on region, age, or sex, nor did they conduct meta-regression. We also note some inconsistencies in their analysis, notably the inclusion of results for mortality from all cardiovascular causes (not strictly IHD) from Hoek et al. [120], as well as errors - assigning identical results to Peters et al. [47] and Ruidavets et al. [48], and including a negative result from Stieb et al. [66], which was not reported by the authors of that study. Our pooled relative risk for time-series studies was also comparable to that of Mills et al. [10] (after converting to ppb), who reported a pooled relative risk of 1.0086 (95% CI 1.0052–1.012) per 10 μg/m^3^ based on results from 10 studies (separate pooled estimates were provided for an additional 11 studies of elderly populations). Limitations of Mills et al.’s review include limited evaluation of sources of heterogeneity or consideration of results from single vs. multi-pollutant models, and failure to assess risk of bias across multiple domains (adjustment for “important confounders” was an inclusion criterion). Other systematic reviews and meta-analyses of the short term association of PM_2.5_ and PM_10_ and IHD morbidity reported pooled effect estimates of comparable magnitude [11, 12].

### Other lines of evidence

We have not conducted a systemic review of toxicological and human clinical evidence. However, in order to inform our conclusions about the existence of a causal association between short term NO_2_ exposure and IHD morbidity, we present a brief summary of evidence evaluating possible pathophysiological mechanisms which could explain the associations observed in epidemiological studies. While the evidence specifically linking NO_2_ to adverse cardiovascular effects in controlled animal toxicological studies is limited, some studies have identified adverse cardiovascular effects specifically from NO_2_ exposure, including increased blood viscosity, red cell rigidity and red cell aggregation after one and 3 months exposure [121], and endothelial dysfunction, oxidative stress and inflammation following 7 day exposure [122]. With respect to effects of mixtures, Selikop et al. reported increased atherosclerosis response indicators (endothelin-1, matrix metalloproteinase-9, tissue inhibitor of metalloproteinase-2, thiobarbituric acid reactive substances) attributed to NO_2_ following 50 day exposure to diesel or gasoline exhaust [123], Zhang et al. reported that co-exposure to NO_2_, SO_2_ and PM_10_ for 28 days resulted in endothelial dysfunction, increased inflammatory response, decreased blood pressure and increased heart rate [124], and Mauderly et al. found that a five gas mixture of NO_2_, SO_2_, CO, NO and NH_3_ for 50 days resulted in increases in endothelin-1, matrix metalloproteinase-9, tissue inhibitor of metalloproteinase-2, heme oxygenase-1 and thiobarbituric acid reactive substances [125]. Studies have also noted persistent adverse effects of diesel emissions after particle filtration [126, 127], potentially implicating gaseous phase emissions, including NO_2_.

Controlled human exposure studies have produced mixed results. Scaife et al. reported no association between NO_2_ exposure and heart rate, heat rate variability (HRV), ectopic beats, or arrhythmias in adults with stable IHD [128], while Huang et al. reported significant associations with HRV in healthy young adults [129]. Riedl et al. found no association with coagulation factors, blood pressure, oxygen saturation or cardiovascular symptom scores in individuals with mild asthma [130] and Langrish et al. reported no significant associations with measures of fibrinolytic function in healthy males [131]. In an in-vitro study, Channell et al. found that exposure to plasma from healthy volunteers exposed to NO_2_ was associated with increased concentrations of intracellular and vascular cell adhesion molecules in human coronary artery endothelial cells [132]. Both Frampton et al. and Posin et al. reported reduced haemoglobin and hematocrit following NO_2_ exposure in healthy adults [133, 134], while Langrish et al. did not [131].

### Overall rating of quality and strength of evidence

In their 2016 Science Assessments, both the US Environmental Protection Agency (EPA) and Health Canada concluded that the evidence was suggestive of, but not sufficient to infer, a causal association between NO_2_ and IHD morbidity, based on a smaller number of studies, and fewer examining the impact of adjustment for co-pollutants than considered here, as well as limited and inconsistent supporting mechanistic evidence from controlled human and animal studies [1, 2]. Our observation that short term exposure to NO_2_ was significantly associated with IHD morbidity based on pooled ORs and RRs from a much larger number of case-crossover and time series studies, the majority of which were rated low or probably low risk of bias across most domains, provides good evidence that short term exposure to air pollution in general and particularly traffic related air pollution triggers IHD morbidity. With respect to the probability of a causal relationship specifically with NO_2_, following the Navigation Guide methodology [135] and the causality determination framework used by the US EPA/Health Canada [2] (Additional Files 11, 12), the significant heterogeneity among studies even after accounting for sources of heterogeneity, the relatively large proportion of studies (46.5%) rated as probably high or high risk of bias due to confounding by temporal cycles and weather, evidence of confounding related to other pollutants, inability to assess confounding from concomitant traffic-related exposures including noise and stress, and apparent publication bias affecting case-crossover studies, are considered downgrading factors in interpreting the overall strength of evidence. In total, 15 case-crossover and time-series studies provided estimates based on both single and multi-pollutant models. Multi-pollutant models should be interpreted with caution in that the sensitivity of the effect of one pollutant to inclusion of other pollutants in a joint model is affected by factors such as the correlation among pollutants and their relative degree of exposure measurement error [136]. Nonetheless, although pooled estimates based on multi-pollutant models were smaller in magnitude than from single pollutant models, the differences between pooled estimates were not statistically significant. Thus, while effects of NO_2_ appear to be confounded by co-pollutants, there is still evidence of an association after accounting for this. In a recent causal-modelling analysis of NO_2_, PM_2.5_ and mortality in 135 US cities, Schwartz et al. concluded that NO_2_ was independently associated with mortality, although residual confounding by other pollutants could not be ruled out [7]. Similarly, in their systematic review and meta-analysis attempting to distinguish effects of particulate matter and NO_2_ on mortality and hospital admissions in time-series studies, Mills et al. concluded that effects of NO_2_ were generally robust to inclusion of particulate matter measures in multi-pollutant models, strengthening the case for a causal relationship [137]. However, their analysis included only five studies of cardiac hospital admissions (not specifically IHD), and they could not rule out residual confounding by primary combustion particles [137]. While in the present review, accounting for publication bias affecting case-crossover studies reduced the magnitude of the pooled OR, the 95% CI still excluded 1 or no effect. In contrast to these downgrading factors, characterization of the exposure response relationship as linear or linear with a threshold in 11 of the 13 studies in which this was evaluated, is considered an upgrading factor, albeit based on a small number of studies. We therefore conclude that the epidemiological evidence suggests that there is a likely causal relationship between short term NO_2_ exposure and IHD morbidity, but important uncertainties remain, particularly related to the contribution of co-pollutants or other concomitant exposures, and the relative lack of supporting evidence from toxicological and controlled human studies. Upgrading to a conclusion that there is sufficient evidence for a causal association would require more conclusive evidence ruling out potential confounders as well as consistent supporting animal toxicological and human clinical evidence. Our conclusion parallels that of Health Canada in its determination that there is a likely causal relationship between short term exposure to NO_2_ and mortality [2], with similar caveats regarding potential confounding and a lack of supporting mechanistic evidence. USEPA differed in its assessment, concluding that the evidence is suggestive of, but not sufficient to infer, a causal relationship between short-term NO2 exposure and mortality [1]. Future time-series and case-crossover studies could address uncertainties related to confounding by co-pollutants by consistently examining effects in multi-pollutant models, recognizing the caveats noted earlier. Since few of the studies we reviewed addressed the shape of the concentration-response relationship, further examination in future studies would also be informative. Novel designs are needed to address other potential traffic-related confounders such as noise and stress. Finally, in order to facilitate evaluation of risk of bias, we recommend greater transparency in reporting on exposure assessment, particularly with respect to the number of ground monitors providing exposure data and proportion of days with missing data, and on specification of covariates in regression models. Consistent reporting of effects based on 24 h average concentrations (in addition to other metrics if desired) would obviate the need to convert effect size estimates from other metrics based on assumptions about the relative magnitude of effect.

## Conclusions

We conducted a synthesis of the evidence from 86 case-crossover and time-series studies examining the association between NO_2_ and IHD morbidity, including sensitivity analyses based on pooling method, leave one out analysis and trim and fill, as well as subgroup analyses and/or meta-regression of single vs. multi-pollutant models and effects of region, age of study, study exposure levels, risk of bias ratings, age and sex. We concluded that there is a likely causal relationship between short term NO_2_ exposure and morbidity from ischemic heart disease, but important uncertainties remain, particularly related to the contribution of co-pollutants or other concomitant exposures, and the limited supporting evidence from animal toxicological studies and controlled human exposure studies.

## Supplementary information

**Additional file 1.** Details of Search Strategies.

**Additional file 2.** Summary of Risk of Bias Criteriaa.

**Additional file 3.** Reasons for Risk of Bias Ratings > Low Risk.

**Additional file 4.** Forest plot of case-crossover studies from Europe and North America (AMI, acute myocardial infarction, NSTEMI, non ST-elevation MI, STEMI, ST-elevation MI, EV, emergency visit, HA, hospital admission, T, temperature, Ox, total oxidants, GSH, glutathione related oxidative potential).

**Additional file 5.** Forest plot of case-crossover studies outside Europe and North America (AMI, acute myocardial infarction, STEMI, ST-elevation MI, EV, emergency visit, HA, hospital admission, T, temperature).

**Additional file 6.** Forest plot of time-series studies from Europe and North America (AMI, acute myocardial infarction, AP, angina pectoris, IHD, ischemic heart disease, STEMI, ST-elevation MI, EV, emergency visit, HA, hospital admission, MD, physician visit, lag reported in days).

**Additional file 7.** Forest plot of time-series studies from outside Europe and North America (AMI, acute myocardial infarction, AP, angina pectoris, IHD, ischemic heart disease, STEMI, ST-elevation MI, EV, emergency visit, HA, hospital admission, lag reported in days).

**Additional file 8.** Sensitivity analyses by estimator.

**Additional file 9.** Leave one out analysis.

**Additional file 10.** Funnel plot of log(Relative Risk) vs. standard error for time-series studies from Fig. 4. Filled circles represent observed values, open circles represent missing studies identified with trim and fill, and the vertical line represents the log of the pooled relative risk. In the absence of publication bias, points should be symmetrically distributed around the vertical line, with smaller studies (larger standard errors on vertical axis) more widely scattered. Filling the plot with points mirroring observed values corrects for apparently missing smaller and/or negative studies which may have been suppressed due to publication bias.

**Additional file 11.** Navigation Guide Criteria for Overall Quality and Strength of Evidence^a^.

**Additional file 12.** USEPA/Health Canada Criteria for Evaluating Likelihood of Causal Relationship^a^.

## Data Availability

The datasets used and/or analysed during the current study are available from the corresponding author on reasonable request.

## Abbreviations

AMI: Acute myocardial infarction
AP: Angina pectoris
CI: Confidence interval
CO: Carbon monoxide
EPA: Environmental protection agency
EV: Emergency visit
HA: Hospital admission
HRV: Heart rate variability
ICD: International classification of diseases
IHD: Ischemic heart disease
MD: Physician visit
MI: Myocardial infarction
NO_2_: Nitrogen dioxide
NSTEMI: Non-ST elevation myocardial infarction
O_3_: Ozone
OR: Odds ratio
PAF: Population attributable fraction
PM_2.5_: Particulate matter with median aerodynamic diameter < 2.5 μm
PM_10_: Particulate matter with median aerodynamic diameter < 10 μm
PRESS: Peer Review of Electronic Search Strategies
PRISMA: Preferred reporting items for systematic reviews and meta-analyses
REML: Restricted maximum likelihood
RR: Relative risk
SO2: Sulphur dioxide
STEMI: ST elevation myocardial infarction
