# Correction to: Systematic review and meta-analysis of case-crossover and time-series studies of short term outdoor nitrogen dioxide exposure and ischemic heart disease morbidity

**DOI:** 10.1186/s12940-020-00636-4

**Published:** 2020-07-24

**Authors:** David M. Stieb, Carine Zheng, Dina Salama, Rania Berjawi, Monica Emode, Robyn Hocking, Ninon Lyrette, Carlyn Matz, Eric Lavigne, Hwashin H. Shin

**Affiliations:** 1 Environmental Health Science and Research Bureau, Health Canada, 420-757 West Hastings St. - Federal Tower, Vancouver, BC V6C 1A1 Canada; 2 School of Epidemiology and Public Health, University of Ottawa, Ottawa, Canada; 3 School of Population and Public Health, University of British Columbia, Vancouver, Canada; 4 Learning, Knowledge and Library Services, Health Canada, Ottawa, Canada; 5 Water and Air Quality Bureau, Health Canada, Ottawa, Canada; 6 Department of Mathematics and Statistics, Queen’s University, Kingston, Canada

**Correction to: Environ Health 2020 May 1; 19: 47**

**https://doi.org/10.1186/s12940-020-00601-1.**

Following publication of the original article [1], the authors identified an error in the last letter of the author name of Rania Berjawi.

Incorrect name:

Rania BerjawI

Correct name:

Rania Berjawi

The author group has been updated above and the original article [1] has been corrected.
