# Supplementary material for: Systematic review and meta-analysis of case-crossover and time-series studies of short term outdoor nitrogen dioxide exposure and ischemic heart disease morbidity
Source: Environ Health. 2020 May 1;19:47. doi: 10.1186/s12940-020-00601-1 (PMC7195719; doi:10.1186/s12940-020-00601-1)
Supplement: Supplementary file 1 — Additional file 1. Details of Search Strategies. [file 12940_2020_601_MOESM1_ESM.docx]

**Additional File 1 – Details of Search Strategies**

Database(s): Ovid MEDLINE(R) Epub Ahead of Print, In-Process & Other Non-Indexed Citations, Ovid MEDLINE(R) Daily, Ovid MEDLINE and Versions(R) 1946 to February 07, 2018. Updated August 27, 2019.

Search Strategy:

| **#** | **Searches** | **Results** |
| --- | --- | --- |
| 1 | exp nitrogen oxides/ | 103678 |
| 2 | ((nitrogen adj2 oxide*) or nitric oxide* or dinitrogen monoxide or dinitrogen oxide or hyponitrous acid anhydride or nitrogen hypoxide or nitrogen monoxide or nitrogen protoxide).tw,kf. | 137450 |
| 3 | (nitrogen dioxide or nitrogen peroxide or "NO_2_" or "NOx").tw,kf. | 20441 |
| 4 | or/1-3 [NOx] | 183579 |
| 5 | ((respira* or lung* or bronch* or cilia* or laryng* or pulmonar* or alveol* or aortic* or vascular* or cerebrovasc* or pericard* or endocard* or pleural or thorax or pneumo* or thoracic or trachea* or cardio* or cardia* or heart? or vascula* or atria* or ventric* or myocard* or coronary) adj2 (health or symptom* or infect* or disease* or disorder* or dysfunction* or syndrome* granuloma* or neoplas* or cancer* or tumor* or tumour* or ischemia* or mortal* or morbid* or fatal* or death? or admission* or hospital* or emergency or emergencies)).tw,kf. | 1061933 |
| 6 | exp Respiratory Tract Infections/ | 332704 |
| 7 | exp Respiratory Tract Diseases/ | 1227094 |
| 8 | (pleural empyema or whooping cough or pertussis or bronchiectasis).tw,kf. | 37905 |
| 9 | ((bronchial adj2 (fistula* or hyperreactiv* or neoplasm* or spasm*)) or bronchitis or bronchiolitis or bronchogenic cyst* or bronchopneumonia* or bronchiectas?s or bronchospasm*).tw,kf. | 52145 |
| 10 | ((laryng* adj2 (edema* or neoplasm* or nerve injur*)) or laryngitis or croup or supraglottitis or tuberculos* or laryngotracheobronchit*).tw,kf. | 203143 |
| 11 | acute chest syndrome*.tw,kf. | 890 |
| 12 | cystic fibrosis.tw,kf. | 39849 |
| 13 | lung abscess*.tw,kf. | 3038 |
| 14 | blastomycos*.tw,kf. | 2306 |
| 15 | pneumonia*.tw,kf. | 146884 |
| 16 | pulmonary aspergillos*.tw,kf. | 2989 |
| 17 | alveolitis.tw,kf. | 4032 |
| 18 | pneumonitis.tw,kf. | 12372 |
| 19 | ((chronic obstructive adj2 (pulmonary or airway or lung)) or chronic airflow obstruction or "COPD" or "COAD" or "AECOPD" or ("OPD" or obstructive pulmonary)).tw,kf. | 58955 |
| 20 | pulmonary edema*.tw,kf. | 13996 |
| 21 | pulmonary embolism*.tw,kf. | 30365 |
| 22 | respiratory distress syndrome*.tw,kf. | 22702 |
| 23 | bronchiogenic carcinoma*.tw,kf. | 110 |
| 24 | pulmonary nodule*.tw,kf. | 5941 |
| 25 | pancoast syndrome.tw,kf. | 134 |
| 26 | pulmonary sclerosing hemangioma*.tw,kf. | 117 |
| 27 | exp Cardiovascular Diseases/ | 2166950 |
| 28 | (NSTEMI or STEMI or cardiogenic shock* or ((cardiovascular or heart or myocardial) adj2 (stroke* or attack* or infarct* or event?)) or ((ischemic* or hemmorhagic or haemmorhagic) adj2 stroke?)).tw,kf. | 252316 |
| 29 | (angina or stenocardi*).tw,kf. | 51051 |
| 30 | (((heart or cardiac or myocardia*) adj2 (failure or decompensat*)) or cardiac edema or paroxysmal dyspnea or cardio-renal syndrome* or cardiorenal syndrome*).tw,kf. | 158514 |
| 31 | (arrhythmia* or dysrhythmia* or bradycard* or brugada syndrome* or commotio cordis or heart block* or long QT syndrome or parasystole or pre-excitation syndrome* or tachycardia* or ((atrial or ventricular) adj2 (fibrillation* or flutter)) or conduction disturbance*).tw,kf. | 203105 |
| 32 | or/5-31 [Health Outcomes] | 3883975 |
| 33 | air/ | 17354 |
| 34 | air pollution/ or environmental exposure/ or inhalation exposure/ or exp occupational exposure/ or smog/ | 146365 |
| 35 | environmental pollution/ | 15338 |
| 36 | exp air pollutants/ | 77603 |
| 37 | (air? adj3 (pollut* or environment* or outdoor* or outside*)).tw,kf. | 30108 |
| 38 | Vehicle Emissions/ | 8668 |
| 39 | Automobile Driving/ | 16898 |
| 40 | Parking Facilities/ | 351 |
| 41 | exp transportation/ | 63060 |
| 42 | ((distanc* or proximit* or close* or near) adj3 (traffic* or road* or highway* or transitway*)).tw,kf. | 2099 |
| 43 | traffic exposure*.tw,kf. | 234 |
| 44 | Power Plants/ | 5806 |
| 45 | (power adj2 plant?).tw,kf. | 5618 |
| 46 | ((motor* or vehic* or diesel* or gasoline* or fossil fuel* or crude oil* or coal? or natural gas or petrol*) adj4 (exhaust? or emission* or pollut* or vapor* or vapour* or volatile* or effluvia* or smoke* or fume? or combust* or haze? or smog* or nitrogen oxide* or NOx or nitrogen dioxide* or NO_2_)).tw,kf. | 11575 |
| 47 | (automobile* or automotive* or autocar* or autobus* or taxi* or engine? or car or cars or truck* or bus or buses or bussing or highway* or high way* or motorway* or motor way or transitway* or road* or parkade* or parking* or carpark* or car park* or traffic or street* or motorcycl* or motorbi* or expressway* or parkway*).tw,kf. | 134145 |
| 48 | or/33-47 [NOx Sources] | 418362 |
| 49 | exp hospitalization/ | 202889 |
| 50 | child hospitalized/ or adolescent hospitalized/ | 6530 |
| 51 | exp patient care/ | 844917 |
| 52 | exp primary health care/ | 134468 |
| 53 | exp hospital departments/ | 166722 |
| 54 | exp hospitals/ | 248647 |
| 55 | health facilities/ | 13600 |
| 56 | ambulatory care facilities/ | 16679 |
| 57 | Physicians' Offices/ | 1685 |
| 58 | (((clinic* or emergen* or doctor* or physician* or pediatric* or paediatric* or cardi* or respir* or healthcare or patient* or medical or primary care or health team or ambulatory care or "walk in") adj4 (admission* or admit* or readmission* or readmit* or discharg* or handoff or hand* off or transfer* or visit? or consult*)) or hospital*).tw,kf. | 1233251 |
| 59 | (("walk in" or clinic* or health care or healthcare or practitioner* or physician* or doctor* or medical or health care) adj3 (appointment* or booking* or office? or meeting*)).tw,kf. | 19373 |
| 60 | ((CVD or STEMI or NSTEMI or "MI" or infarct* or cardio* or cardia* or myocard* or pericard* or coronary or respirat* or pulmonary or heart? or vascula* or atria* or ventric*) adj3 (event or events or inciden* or risk? or crisis or crises or symptom* or emergencies or emergency)).tw,kf. | 298005 |
| 61 | (primary adj2 care).tw,kf. | 118122 |
| 62 | exp morbidity/ | 475798 |
| 63 | exp mortality/ | 337377 |
| 64 | exp epidemiology/ | 24531 |
| 65 | epidemiological monitoring/ | 5805 |
| 66 | (IQR or interquartile range* or inter quartile range* or mortalit* or morbidit* or epidemiolog* or death* or dead* or fatal* or lethal*).tw,kf. | 1889892 |
| 67 | or/49-66 [Hospitalization, Recorded Health Events] | 4202430 |
| 68 | 4 and 32 and 48 and 67 [NOx + Health + NOx Sources + Hospitalization] | 2220 |

Database(s): Embase 1974 to 2018 February 13

Search Strategy:

| **#** | **Searches** | **Results** |
| --- | --- | --- |
| 1 | nitrogen oxide/ or nitrogen dioxide/ | 19125 |
| 2 | ((nitrogen adj2 oxide*) or nitric oxide* or dinitrogen monoxide or dinitrogen oxide or hyponitrous acid anhydride or nitrogen hypoxide or nitrogen monoxide or nitrogen protoxide).tw,kw. | 176192 |
| 3 | (nitrogen dioxide or nitrogen peroxide or "NO_2_" or "NOx").tw,kw. | 32286 |
| 4 | or/1-3 [NOx] | 206118 |
| 5 | ((respira* or lung* or bronch* or cilia* or laryng* or pulmonar* or alveol* or aortic* or vascular* or cerebrovasc* or pericard* or endocard* or pleural or thorax or pneumo* or thoracic or trachea* or cardio* or cardia* or heart? or vascula* or atria* or ventric* or myocard* or coronary) adj2 (health or symptom* or infect* or disease* or disorder* or dysfunction* or syndrome* granuloma* or neoplas* or cancer* or tumor* or tumour* or ischemia* or mortal* or morbid* or fatal* or death? or admission* or hospital* or emergency or emergencies)).tw,kw. | 1493275 |
| 6 | respiratory tract disease/ or acute respiratory tract disease/ or exp bronchus disease/ or chronic aspecific respiratory tract disease/ or chronic respiratory tract disease/ or exp larynx disorder/ or exp lung disease/ or exp mediastinum disease/ or exp pleura disease/ or exp respiratory distress/ or exp respiratory failure/ or exp respiratory function disorder/ or exp respiratory tract hemorrhage/ or exp respiratory tract infection/ or exp respiratory tract inflammation/ or exp respiratory tract tumor/ or exp trachea disease/ or exp tracheobronchomalacia/ or obstructive airway disease/ or airway constriction/ or airway obstruction/ or exp bronchitis/ or bronchus obstruction/ or bronchus stenosis/ or chronic obstructive lung disease/ or exp lung emphysema/ or trachea stenosis/ | 2029890 |
| 7 | (pleural empyema or whooping cough or pertussis or bronchiectasis).tw,kw. | 43256 |
| 8 | ((bronchial adj2 (fistula* or hyperreactiv* or neoplasm* or spasm*)) or bronchitis or bronchiolitis or bronchogenic cyst* or bronchopneumonia* or bronchiectas?s or bronchospasm*).tw,kw. | 68850 |
| 9 | ((laryng* adj2 (edema* or neoplasm* or nerve injur*)) or laryngitis or croup or supraglottitis or tuberculos* or laryngotracheobronchit*).tw,kw. | 194835 |
| 10 | acute chest syndrome*.tw,kw. | 1694 |
| 11 | cystic fibrosis.tw,kw. | 57233 |
| 12 | lung abscess*.tw,kw. | 2634 |
| 13 | blastomycos*.tw,kw. | 2079 |
| 14 | pneumonia*.tw,kw. | 199482 |
| 15 | pulmonary aspergillos*.tw,kw. | 4048 |
| 16 | alveolitis.tw,kw. | 5461 |
| 17 | pneumonitis.tw,kw. | 19423 |
| 18 | ((chronic obstructive adj2 (pulmonary or airway or lung)) or chronic airflow obstruction or "COPD" or "COAD" or "AECOPD" or ("OPD" or obstructive pulmonary)).tw,kw. | 101298 |
| 19 | pulmonary edema*.tw,kw. | 19376 |
| 20 | pulmonary embolism*.tw,kw. | 45158 |
| 21 | respiratory distress syndrome*.tw,kw. | 32110 |
| 22 | bronchiogenic carcinoma*.tw,kw. | 98 |
| 23 | pulmonary nodule*.tw,kw. | 9374 |
| 24 | pulmonary nodule*.tw,kw. | 9374 |
| 25 | pulmonary sclerosing hemangioma*.tw,kw. | 158 |
| 26 | cardiovascular disease/ or exp cardiovascular infection/ or exp cardiovascular inflammation/ or cardiovascular symptom/ or exp cardiovascular system tumor/ or exp heart disease/ or exp vascular disease/ | 3244135 |
| 27 | (NSTEMI or STEMI or cardiogenic shock* or ((cardiovascular or heart or myocardial) adj2 (stroke* or attack* or infarct* or event?)) or ((ischemic* or hemmorhagic or haemmorhagic) adj2 stroke?)).tw,kw. | 378215 |
| 28 | (angina or stenocardi*).tw,kw. | 70011 |
| 29 | (((heart or cardiac or myocardia*) adj2 (failure or decompensat*)) or cardiac edema or paroxysmal dyspnea or cardio-renal syndrome* or cardiorenal syndrome*).tw,kw. | 251260 |
| 30 | (arrhythmia* or dysrhythmia* or bradycard* or brugada syndrome* or commotio cordis or heart block* or long QT syndrome or parasystole or pre-excitation syndrome* or tachycardia* or ((atrial or ventricular) adj2 (fibrillation* or flutter)) or conduction disturbance*).tw,kw. | 298942 |
| 31 | or/5-30 [Health Outcomes] | 5347836 |
| 32 | exp air pollution/ or environmental exposure/ or occupational exposure/ | 283288 |
| 33 | (air? adj3 (pollut* or environment* or outdoor* or outside*)).tw,kw. | 42842 |
| 34 | "traffic and transport"/ or exp aviation/ or exp motor vehicle/ or railway/ or ship/ or exp traffic/ | 190113 |
| 35 | ((distanc* or proximit* or close* or near) adj3 (traffic* or road* or highway* or transitway*)).tw,kw. | 2959 |
| 36 | traffic exposure*.tw,kw. | 318 |
| 37 | electric power plant/ | 7506 |
| 38 | (power adj2 plant?).tw,kw. | 9194 |
| 39 | ((motor* or vehic* or diesel* or gasoline* or fossil fuel* or crude oil* or coal? or natural gas or petrol*) adj4 (exhaust? or emission* or pollut* or vapor* or vapour* or volatile* or effluvia* or smoke* or fume? or combust* or haze? or smog* or nitrogen oxide* or NOx or nitrogen dioxide* or NO_2_)).tw,kw. | 18162 |
| 40 | (automobile* or automotive* or autocar* or autobus* or taxi* or engine? or car or cars or truck* or bus or buses or bussing or highway* or high way* or motorway* or motor way or transitway* or road* or parkade* or parking* or carpark* or car park* or traffic or street* or motorcycl* or motorbi* or expressway* or parkway*).tw,kw. | 171949 |
| 41 | or/32-40 [NOx Sources] | 573696 |
| 42 | hospitalization/ or hospitalized adolescent/ or hospitalized child/ or hospitalized infant/ | 300849 |
| 43 | exp medical care/ | 941022 |
| 44 | exp ambulatory care/ | 45694 |
| 45 | health care facility/ or air medical transport/ or exp ambulance/ or health center/ or exp hospital/ or exp isolation facility/ or pain clinic/ or exp pharmacy/ or secondary care center/ or tertiary care center/ | 1157185 |
| 46 | (((clinic* or emergen* or doctor* or physician* or pediatric* or paediatric* or cardi* or respir* or healthcare or patient* or medical or primary care or health team or ambulatory care or "walk in") adj4 (admission* or admit* or readmission* or readmit* or discharg* or handoff or hand* off or transfer* or visit? or consult*)) or hospital*).tw,kw. | 1824442 |
| 47 | (("walk in" or clinic* or health care or healthcare or practitioner* or physician* or doctor* or medical or health care) adj3 (appointment* or booking* or office? or meeting*)).tw,kw. | 27385 |
| 48 | ((CVD or STEMI or NSTEMI or "MI" or infarct* or cardio* or cardia* or myocard* or pericard* or coronary or respirat* or pulmonary or heart? or vascula* or atria* or ventric*) adj3 (event or events or inciden* or risk? or crisis or crises or symptom* or emergencies or emergency)).tw,kw. | 454572 |
| 49 | (primary adj2 care).tw,kw. | 150558 |
| 50 | epidemiological data/ or exp morbidity/ or exp mortality/ or epidemiological monitoring/ or epidemiology/ | 1258073 |
| 51 | (IQR or interquartile range* or inter quartile range* or mortalit* or morbidit* or epidemiolog* or death* or dead* or fatal* or lethal*).tw,kw. | 2577568 |
| 52 | or/42-51 [Hospitalization, Recorded Health Events] | 5718205 |
| 53 | 4 and 31 and 41 and 52 [NOx + Health + NOx Sources + Hospitalization] | 2941 |

Database(s): Global Health 1973 to 2018 Week 05

Search Strategy:

| **#** | **Searches** | **Results** |
| --- | --- | --- |
| 1 | exp nitrogen oxides/ | 16898 |
| 2 | ((nitrogen adj2 oxide*) or nitric oxide* or dinitrogen monoxide or dinitrogen oxide or hyponitrous acid anhydride or nitrogen hypoxide or nitrogen monoxide or nitrogen protoxide).tw. | 22301 |
| 3 | (nitrogen dioxide or nitrogen peroxide or "NO_2_" or "NOx").tw. | 5264 |
| 4 | or/1-3 [NOx] | 26619 |
| 5 | ((respira* or lung* or bronch* or cilia* or laryng* or pulmonar* or alveol* or aortic* or vascular* or cerebrovasc* or pericard* or endocard* or pleural or thorax or pneumo* or thoracic or trachea* or cardio* or cardia* or heart? or vascula* or atria* or ventric* or myocard* or coronary) adj2 (health or symptom* or infect* or disease* or disorder* or dysfunction* or syndrome* granuloma* or neoplas* or cancer* or tumor* or tumour* or ischemia* or mortal* or morbid* or fatal* or death? or admission* or hospital* or emergency or emergencies)).tw. | 199570 |
| 6 | exp respiratory diseases/ | 105799 |
| 7 | (pleural empyema or whooping cough or pertussis or bronchiectasis).tw. | 7605 |
| 8 | ((bronchial adj2 (fistula* or hyperreactiv* or neoplasm* or spasm*)) or bronchitis or bronchiolitis or bronchogenic cyst* or bronchopneumonia* or bronchiectas?s or bronchospasm*).tw. | 8132 |
| 9 | ((laryng* adj2 (edema* or neoplasm* or nerve injur*)) or laryngitis or croup or supraglottitis or tuberculos* or laryngotracheobronchit*).tw. | 53261 |
| 10 | acute chest syndrome*.tw. | 74 |
| 11 | cystic fibrosis.tw. | 5687 |
| 12 | lung abscess*.tw. | 317 |
| 13 | blastomycos*.tw. | 9532 |
| 14 | pneumonia*.tw. | 57693 |
| 15 | pulmonary aspergillos*.tw. | 1856 |
| 16 | alveolitis.tw. | 614 |
| 17 | pneumonitis.tw. | 1756 |
| 18 | ((chronic obstructive adj2 (pulmonary or airway or lung)) or chronic airflow obstruction or "COPD" or "COAD" or "AECOPD" or ("OPD" or obstructive pulmonary)).tw. | 7657 |
| 19 | pulmonary edema*.tw. | 652 |
| 20 | pulmonary embolism*.tw. | 971 |
| 21 | respiratory distress syndrome*.tw. | 2439 |
| 22 | bronchiogenic carcinoma*.tw. | 2 |
| 23 | pulmonary nodule*.tw. | 389 |
| 24 | pancoast syndrome.tw. | 8 |
| 25 | pulmonary sclerosing hemangioma*.tw. | 5 |
| 26 | exp cardiovascular diseases/ or cardiovascular disorders/ | 99416 |
| 27 | (NSTEMI or STEMI or cardiogenic shock* or ((cardiovascular or heart or myocardial) adj2 (stroke* or attack* or infarct* or event?)) or ((ischemic* or hemmorhagic or haemmorhagic) adj2 stroke?)).tw. | 17599 |
| 28 | (angina or stenocardi*).tw. | 2216 |
| 29 | (((heart or cardiac or myocardia*) adj2 (failure or decompensat*)) or cardiac edema or paroxysmal dyspnea or cardio-renal syndrome* or cardiorenal syndrome*).tw. | 7725 |
| 30 | (arrhythmia* or dysrhythmia* or bradycard* or brugada syndrome* or commotio cordis or heart block* or long QT syndrome or parasystole or pre-excitation syndrome* or tachycardia* or ((atrial or ventricular) adj2 (fibrillation* or flutter)) or conduction disturbance*).tw. | 7400 |
| 31 | or/5-30 [Health Outcomes] | 338880 |
| 32 | air pollution/ or exp air pollutants/ | 18643 |
| 33 | (air? adj3 (pollut* or environment* or outdoor* or outside*)).tw. | 20378 |
| 34 | vehicle emissions/ | 3 |
| 35 | exp vehicles/ | 5771 |
| 36 | parking areas/ or motoring/ | 49 |
| 37 | ((distanc* or proximit* or close* or near) adj3 (traffic* or road* or highway* or transitway*)).tw. | 1014 |
| 38 | traffic exposure*.tw. | 146 |
| 39 | (power adj2 plant?).tw. | 1875 |
| 40 | ((motor* or vehic* or diesel* or gasoline* or fossil fuel* or crude oil* or coal? or natural gas or petrol*) adj4 (exhaust? or emission* or pollut* or vapor* or vapour* or volatile* or effluvia* or smoke* or fume? or combust* or haze? or smog* or nitrogen oxide* or NOx or nitrogen dioxide* or NO_2_)).tw. | 2921 |
| 41 | (automobile* or automotive* or autocar* or autobus* or taxi* or engine? or car or cars or truck* or bus or buses or bussing or highway* or high way* or motorway* or motor way or transitway* or road* or parkade* or parking* or carpark* or car park* or traffic or street* or motorcycl* or motorbi* or expressway* or parkway*).tw. | 28217 |
| 42 | or/32-41 [NOx Sources] | 50963 |
| 43 | hospital stay/ or hospital admission/ or hospital care/ | 11445 |
| 44 | primary health care/ | 10072 |
| 45 | health centres/ or hospitals/ | 35142 |
| 46 | (((clinic* or emergen* or doctor* or physician* or pediatric* or paediatric* or cardi* or respir* or healthcare or patient* or medical or primary care or health team or ambulatory care or "walk in") adj4 (admission* or admit* or readmission* or readmit* or discharg* or handoff or hand* off or transfer* or visit? or consult*)) or hospital*).tw. | 237731 |
| 47 | (("walk in" or clinic* or health care or healthcare or practitioner* or physician* or doctor* or medical or health care) adj3 (appointment* or booking* or office? or meeting*)).tw. | 2385 |
| 48 | ((CVD or STEMI or NSTEMI or "MI" or infarct* or cardio* or cardia* or myocard* or pericard* or coronary or respirat* or pulmonary or heart? or vascula* or atria* or ventric*) adj3 (event or events or inciden* or risk? or crisis or crises or symptom* or emergencies or emergency)).tw. | 53310 |
| 49 | (primary adj2 care).tw. | 22073 |
| 50 | morbidity/ or mortality/ | 96216 |
| 51 | epidemiology/ | 250285 |
| 52 | (IQR or interquartile range* or inter quartile range* or mortalit* or morbidit* or epidemiolog* or death* or dead* or fatal* or lethal*).tw. | 531399 |
| 53 | or/43-52 [Hospitalization, Recorded Health Events] | 723805 |
| 54 | 4 and 31 and 42 and 53 [NOx + Health + NOx Sources + Hospitalization] | 1184 |

Database(s): EBM Reviews - Cochrane Central Register of Controlled Trials January 2018

Search Strategy:

| **#** | **Searches** | **Results** |
| --- | --- | --- |
| 1 | exp nitrogen oxides/ | 3079 |
| 2 | ((nitrogen adj2 oxide*) or nitric oxide* or dinitrogen monoxide or dinitrogen oxide or hyponitrous acid anhydride or nitrogen hypoxide or nitrogen monoxide or nitrogen protoxide).tw,kf. | 4826 |
| 3 | (nitrogen dioxide or nitrogen peroxide or "NO_2_" or "NOx").tw,kf. | 473 |
| 4 | or/1-3 [NOx] | 6528 |
| 5 | ((respira* or lung* or bronch* or cilia* or laryng* or pulmonar* or alveol* or aortic* or vascular* or cerebrovasc* or pericard* or endocard* or pleural or thorax or pneumo* or thoracic or trachea* or cardio* or cardia* or heart? or vascula* or atria* or ventric* or myocard* or coronary) adj2 (health or symptom* or infect* or disease* or disorder* or dysfunction* or syndrome* granuloma* or neoplas* or cancer* or tumor* or tumour* or ischemia* or mortal* or morbid* or fatal* or death? or admission* or hospital* or emergency or emergencies)).tw,kf. | 82383 |
| 6 | exp Respiratory Tract Infections/ | 10042 |
| 7 | exp Respiratory Tract Diseases/ | 41598 |
| 8 | (pleural empyema or whooping cough or pertussis or bronchiectasis).tw,kf. | 1737 |
| 9 | ((bronchial adj2 (fistula* or hyperreactiv* or neoplasm* or spasm*)) or bronchitis or bronchiolitis or bronchogenic cyst* or bronchopneumonia* or bronchiectas?s or bronchospasm*).tw,kf. | 4694 |
| 10 | ((laryng* adj2 (edema* or neoplasm* or nerve injur*)) or laryngitis or croup or supraglottitis or tuberculos* or laryngotracheobronchit*).tw,kf. | 3914 |
| 11 | acute chest syndrome*.tw,kf. | 98 |
| 12 | cystic fibrosis.tw,kf. | 3705 |
| 13 | lung abscess*.tw,kf. | 35 |
| 14 | blastomycos*.tw,kf. | 6 |
| 15 | pneumonia*.tw,kf. | 7509 |
| 16 | pulmonary aspergillos*.tw,kf. | 54 |
| 17 | alveolitis.tw,kf. | 95 |
| 18 | pneumonitis.tw,kf. | 771 |
| 19 | ((chronic obstructive adj2 (pulmonary or airway or lung)) or chronic airflow obstruction or "COPD" or "COAD" or "AECOPD" or ("OPD" or obstructive pulmonary)).tw,kf. | 12648 |
| 20 | pulmonary edema*.tw,kf. | 489 |
| 21 | pulmonary embolism*.tw,kf. | 1975 |
| 22 | respiratory distress syndrome*.tw,kf. | 2162 |
| 23 | bronchiogenic carcinoma*.tw,kf. | 0 |
| 24 | pulmonary nodule*.tw,kf. | 143 |
| 25 | pancoast syndrome.tw,kf. | 0 |
| 26 | pulmonary sclerosing hemangioma*.tw,kf. | 0 |
| 27 | exp Cardiovascular Diseases/ | 78173 |
| 28 | (NSTEMI or STEMI or cardiogenic shock* or ((cardiovascular or heart or myocardial) adj2 (stroke* or attack* or infarct* or event?)) or ((ischemic* or hemmorhagic or haemmorhagic) adj2 stroke?)).tw,kf. | 30219 |
| 29 | (angina or stenocardi*).tw,kf. | 8324 |
| 30 | (((heart or cardiac or myocardia*) adj2 (failure or decompensat*)) or cardiac edema or paroxysmal dyspnea or cardio-renal syndrome* or cardiorenal syndrome*).tw,kf. | 16229 |
| 31 | (arrhythmia* or dysrhythmia* or bradycard* or brugada syndrome* or commotio cordis or heart block* or long QT syndrome or parasystole or pre-excitation syndrome* or tachycardia* or ((atrial or ventricular) adj2 (fibrillation* or flutter)) or conduction disturbance*).tw,kf. | 16928 |
| 32 | or/5-31 [Health Outcomes] | 202306 |
| 33 | air/ | 497 |
| 34 | air pollution/ or environmental exposure/ or inhalation exposure/ or exp occupational exposure/ or smog/ | 1152 |
| 35 | environmental pollution/ | 11 |
| 36 | exp air pollutants/ | 481 |
| 37 | (air? adj3 (pollut* or environment* or outdoor* or outside*)).tw,kf. | 522 |
| 38 | Vehicle Emissions/ | 71 |
| 39 | Automobile Driving/ | 730 |
| 40 | Parking Facilities/ | 2 |
| 41 | exp transportation/ | 595 |
| 42 | ((distanc* or proximit* or close* or near) adj3 (traffic* or road* or highway* or transitway*)).tw,kf. | 45 |
| 43 | traffic exposure*.tw,kf. | 5 |
| 44 | Power Plants/ | 22 |
| 45 | (power adj2 plant?).tw,kf. | 16 |
| 46 | ((motor* or vehic* or diesel* or gasoline* or fossil fuel* or crude oil* or coal? or natural gas or petrol*) adj4 (exhaust? or emission* or pollut* or vapor* or vapour* or volatile* or effluvia* or smoke* or fume? or combust* or haze? or smog* or nitrogen oxide* or NOx or nitrogen dioxide* or NO_2_)).tw,kf. | 207 |
| 47 | (automobile* or automotive* or autocar* or autobus* or taxi* or engine? or car or cars or truck* or bus or buses or bussing or highway* or high way* or motorway* or motor way or transitway* or road* or parkade* or parking* or carpark* or car park* or traffic or street* or motorcycl* or motorbi* or expressway* or parkway*).tw,kf. | 3379 |
| 48 | or/33-47 [NOx Sources] | 6458 |
| 49 | exp hospitalization/ | 11743 |
| 50 | child hospitalized/ or adolescent hospitalized/ | 107 |
| 51 | exp patient care/ | 36584 |
| 52 | exp primary health care/ | 4240 |
| 53 | exp hospital departments/ | 3022 |
| 54 | exp hospitals/ | 3107 |
| 55 | health facilities/ | 62 |
| 56 | ambulatory care facilities/ | 403 |
| 57 | Physicians' Offices/ | 22 |
| 58 | (((clinic* or emergen* or doctor* or physician* or pediatric* or paediatric* or cardi* or respir* or healthcare or patient* or medical or primary care or health team or ambulatory care or "walk in") adj4 (admission* or admit* or readmission* or readmit* or discharg* or handoff or hand* off or transfer* or visit? or consult*)) or hospital*).tw,kf. | 109946 |
| 59 | (("walk in" or clinic* or health care or healthcare or practitioner* or physician* or doctor* or medical or health care) adj3 (appointment* or booking* or office? or meeting*)).tw,kf. | 6315 |
| 60 | ((CVD or STEMI or NSTEMI or "MI" or infarct* or cardio* or cardia* or myocard* or pericard* or coronary or respirat* or pulmonary or heart? or vascula* or atria* or ventric*) adj3 (event or events or inciden* or risk? or crisis or crises or symptom* or emergencies or emergency)).tw,kf. | 42028 |
| 61 | (primary adj2 care).tw,kf. | 12981 |
| 62 | exp morbidity/ | 13547 |
| 63 | exp mortality/ | 11696 |
| 64 | exp epidemiology/ | 43 |
| 65 | epidemiological monitoring/ | 29 |
| 66 | (IQR or interquartile range* or inter quartile range* or mortalit* or morbidit* or epidemiolog* or death* or dead* or fatal* or lethal*).tw,kf. | 94834 |
| 67 | or/49-66 [Hospitalization, Recorded Health Events] | 246028 |
| 68 | 4 and 32 and 48 and 67 [NOx + Health + NOx Sources + Hospitalization] | 51 |

Toxline (1900-2018)

| **Search** | **Database** | **Query** | **Time** | **Result** |
| --- | --- | --- | --- | --- |
| # 5 | toxline | ( #1 AND #2 AND #3 AND #4 ) AND NOT PubMed [org] AND NOT pubdart [org] | 09:12:37 | 39 |
| # 4 | toxline | ( "nitrogen dioxide" OR "stikstofdioxyde dutch " "stickstoffdioxid german " "nitrogen peroxide" OR nitrito OR "azoto italian " "azote french " OR 10102-44-0 [rn] ) [not] PubMed [org] [not] pubdart [org] | 09:10:17 | 5874 |
| # 3 | toxline | ( exhaust OR emission OR pollute OR pollutant OR pollution OR automobile OR automotive OR autocar OR autobus OR taxi OR engine OR car OR cars OR truck OR bus OR buses OR bussing OR highway OR "high way" OR motorway OR "motor way" OR transitway OR road OR parkade OR parking OR carpark OR "car park" OR traffic OR street OR motorcycle OR motorbike OR expressway OR parkway OR "power plant" ) AND NOT PubMed [org] AND NOT pubdart [org] | 09:08:33 | 500000 |
| # 2 | toxline | ( clinic OR emergency OR emergencies OR doctor OR physician OR healthcare OR patient OR medical OR "primary care" OR "health team" OR "ambulatory care" OR "walk in" OR hospital OR event OR incident OR risk OR crisis OR crises ) AND NOT PubMed [org] AND NOT pubdart [org] | 09:06:13 | 500000 |
| # 1 | toxline | ( respiratory OR lung OR bronchial OR cilia OR laryngeal OR pulmonary OR alveola OR aortic OR vascular OR cerebrovascular OR pericardial OR endocardial OR pleural OR thorax OR thoracic OR tracheal OR cardiopulmonary OR cardiovascular OR cardiac OR heart OR atrial OR ventricle OR ventricular OR myocardial OR coronary ) AND NOT PubMed [org] AND NOT pubdart [org] | 09:04:57 | 500000 |
