# Supplementary material for: Systematic review and meta-analysis of case-crossover and time-series studies of short term outdoor nitrogen dioxide exposure and ischemic heart disease morbidity
Source: Environ Health. 2020 May 1;19:47. doi: 10.1186/s12940-020-00601-1 (PMC7195719; doi:10.1186/s12940-020-00601-1)
Supplement: Supplementary file 2 — Additional file 2. Summary of Risk of Bias Criteriaa. [file 12940_2020_601_MOESM2_ESM.docx]

Additional File 2 - Summary of Risk of Bias Criteria^a^

| Risk of Bias Domain | | Risk of Bias Rating | | | | | | |
| --- | --- | --- | --- | --- | --- | --- | --- | --- |
|  |  | Low | | Probably low | Probably High | | | High |
| Exposure Assessment | | Multiple government or other quality controlled monitors, explicitly <25% missing data | | Multiple government or other quality controlled monitors, assumed <25% missing data | Single government or other quality controlled monitor, or multiple monitors and ≥25% missing data | | | Single government or other quality controlled monitor and ≥25% missing data OR non quality controlled OR source of data not specified |
|  | | Modeling methods supported by evidence of quality, including good quality data inputs, validation against area-based air measurement, or other evidence of the accuracy of data inputs and models not affected by factors which would reduce model accuracy. | | Some evidence against impact of factors which would reduce model accuracy. | Some evidence of impact of factors which would reduce model accuracy. | | | Model previously demonstrated to be unable to describe air levels of exposure for assigning exposure in a research situation OR  Sufficient evidence of impact of factors which would reduce model accuracy. |
| Outcome Assessment | | Vital statistics or hospital data coded according to ICD or based on validated clinical/ laboratory criteria OR physician office data coded according to ICD or based on validated clinical/ laboratory criteria with validation sub-study or other supporting sensitivity analysis | | Physician office data coded according to ICD or based on validated clinical/ laboratory criteria OR Vital statistics, hospital, physician office data or patient self-report not ICD coded or based on clinical laboratory criteria with validation sub-study or other supporting sensitivity analysis | Vital statistics, hospital or physician office data not coded according to ICD nor based on validated clinical/ laboratory criteria | | | Patient self-report OR source poorly documented |
| Confounding  Time-series | | All important confounders accounted for and appropriately specified (or justified that potential confounders were evaluated and omitted or other specifications selected based on sensitivity analysis):   - Time (seasonal cycles and trends) using parametric non-linear function (e.g. natural spline, cubic spline, b-spline, Fourier series) or linear filter (e.g. Shumway filter) - Weather (Temperature and other) using parametric non-linear function (e.g. natural spline, cubic spline, b-spline, polynomial function) or categorical variable(s) - Day of week - Holidays - Influenza epidemics | | Most important confounders accounted for and appropriately specified and those omitted not expected to introduce substantial bias  e.g. Linear adjustment for time and /or weather without justification but judged to be adequate  AND/OR  Adjustment for time using month as covariate  AND/OR  Lacking adjustment for holidays and/or influenza epidemics | Some important confounders accounted for and appropriately specified and those omitted expected to introduce substantial bias  e.g. Linear or non-parametric non-linear (e.g. LOESS, penalized spline, smoothing splines) adjustment for time and/or weather without justification and judged to be inadequate  AND/OR  Lacking adjustment for day of week | | | Did not account for multiple important confounders  e.g. no adjustment for time or weather |
| Case-crossover | - Time - Bidirectional referent day selection + time stratification (Referent day selection stratified by year and month (plus one or more of : day of week, temperature) - Weather (same as time-series) | | e.g. Linear adjustment for weather without justification but judged to be adequate  AND/OR  Lacking adjustment for holidays and/or influenza epidemics | | | e.g. Lacking time stratification by day of week without justification  AND/OR  Linear or non-parametric non-linear (e.g. LOESS, penalized spline, smoothing splines) adjustment for weather without justification and judged to be inadequate  AND/OR  Conditional logistic regression or stratified Cox model in SAS with “ties = discrete” option^b^ | e.g. Unidirectional referent day selection  OR  No adjustment for weather | |
| Completeness of Outcome Data | No missing outcome data or missing data not expected to introduce substantial bias | | Indirect evidence that outcome data complete | | | Indirect evidence that outcome data incomplete | Reason for missing outcome data likely to be related to true outcome, with either imbalance in numbers or reasons for missing data across exposure groups;  Attrition or missing outcome data unbalanced in numbers across exposure groups, with dis-similar reasons for missing data across groups;  Proportion of missing outcomes compared with observed event risk enough to induce biologically relevant bias in effect estimate | |
| Selective Outcome Reporting | All pre-specified (primary and secondary) outcomes outlined in the protocol, methods, abstract, and/or introduction reported completely in the pre-specified way and no outcomes reported that were not pre-specified | | Indirect evidence suggests study was free of selective reporting (e.g. based on study design or nature of outcomes) | | | Indirect evidence suggests study was not free of selective reporting (e.g. based on study design or nature of outcomes) | Not all pre-specified (primary and secondary) outcomes outlined in the protocol, methods, abstract, and/or introduction reported completely in the pre-specified way or outcomes reported that were not pre-specified | |
| Conflict of interest | Study did not receive support from a company, study author, or other entity having a financial interest in the outcome of the study. Examples include the following:   - Funding source is limited to government, non-profit organizations, or academic grants funded by government, foundations and/or non-profit organizations; - Chemicals or other treatment used in study were purchased from a supplier; - Company affiliated staff are not mentioned in the acknowledgements section; - Authors were not employees of a company with a financial interest in the outcome of the study; - Company with a financial interest in the outcome of the study was not involved in the design, conduct, analysis, or reporting of the study and authors had complete access to the data; - Study authors make a claim denying conflicts of interest; - Study authors are unaffiliated with companies with financial interest, and there is no reason to believe a conflict of interest exists; - All study authors are affiliated with a government agency (are prohibited from involvement in projects for which there is a conflict of interest or an appearance of conflict of interest). | | Indirect evidence suggests study was free of conflict | | | Indirect evidence suggests study was not free of conflict | The study received support from a company, study author, or other entity having a financial interest in the outcome of the study. Examples of support include:   - Research funds; - Chemicals, equipment or testing provided at no cost; - Writing services; - Author/staff from study was employee or otherwise affiliated with company with financial interest; - Company limited author access to the data; - Company was involved in the design, conduct, analysis, or reporting of the study; - Study authors claim a conflict of interest | |
| Other | The study was not affected by factors listed under high risk of bias. | | Indirect evidence suggests study was free of other sources of bias. | | | Indirect evidence suggests study was not free of other sources of bias | The study was:   - Affected by a potential source of bias related to the specific study design used; or - Stopped early due to some data-dependent process (including a formal-stopping rule); or - Conducted such that it was affected by interim results (e.g. recruiting additional participants from a subgroup showing greater or lesser effect); or - Claimed to have been fraudulent; or - Affected by some other problem | |

Abbreviations: International Classification of Diseases (ICD); Locally Estimated Polynomial Regression (LOESS)

^a^Reproduced and adapted from Lam J, Sutton P, Kalkbrenner A, et al. A Systematic Review and Meta-Analysis of Multiple Airborne Pollutants and Autism Spectrum Disorder. PLOS ONE. 2016;11(9):e0161851.

^b^Wang SV, Coull BA, Schwartz J, Mittleman MA, Wellenius GA. Potential for bias in case-crossover studies with shared exposures analyzed using SAS. Am J Epidemiol. 2011;174:118-24.
