# Supplementary material for: Systematic review and meta-analysis of case-crossover and time-series studies of short term outdoor nitrogen dioxide exposure and ischemic heart disease morbidity
Source: Environ Health. 2020 May 1;19:47. doi: 10.1186/s12940-020-00601-1 (PMC7195719; doi:10.1186/s12940-020-00601-1)
Supplement: Supplementary file 3 — Additional file 3. Reasons for Risk of Bias Ratings > Low Risk. [file 12940_2020_601_MOESM3_ESM.docx]

Additional File 3 – Reasons for Risk of Bias Ratings > Low Risk

| Study | Design | Reason for Risk of Bias Rating > low | | | | | |
| --- | --- | --- | --- | --- | --- | --- | --- |
|  |  |  | Exposure Assessment | Confounding | Outcome Assessment | Completeness of Outcome Data | Conflict of interest |
| Akbarzadeh 2018 [53] | Case-crossover |  | multiple monitors, assumed<25% missing | unidirectional control selection, weather specification not described |  |  |  |
| Argacha 2016 [36] | Case-crossover |  | multiple monitors, assumed <25% missing data | temperature specified as linear without justification |  |  |  |
| Bard 2014 [37] | Case-crossover |  |  | weather specification not described |  |  |  |
| Barnett 2006 [54] | Case-crossover |  | multiple monitors, assumed <25% missing data | weather specification not described |  |  |  |
| Basu 2012 [30] | Case-crossover |  | multiple monitors, assumed<25% missing | no adjustment for holidays |  |  |  |
| Berglind 2010 [38] | Case-crossover |  |  | no adjustment for holidays, influenza |  |  |  |
| Bhaskaran 2011 [39] | Case-crossover |  |  |  |  |  |  |
| Buszman 2018 [40] | Case-crossover |  | number of monitors not specified | unidirectional referent selection |  |  |  |
| Butland 2016 [41] | Case-crossover |  | correlation between modelled, monitored NO_2_ only moderate (0.54) |  |  |  | 1st author disclosed financial conflicts related to oil and gas industry |
| Cheng 2009 [55] | Case-crossover |  | multiple monitors, assumed <25% missing data | specification of weather variables not described |  |  |  |
| Collart 2015 [42] | Case-crossover |  | multiple monitors, assumed <25% missing | linear specification of weather without justification, no adjustment for holidays, influenza |  |  |  |
| D'Ippoliti 2003 [43] | Case-crossover |  |  | no adjustment for holidays, influenza |  |  |  |
| Evans 2017 [31] | Case-crossover |  | single monitor, assume <25% missing data | no adjustment for holidays, influenza |  |  |  |
| Franck 2014 [56] | Case-crossover |  |  | specification of weather variables not described |  |  |  |
| Hsieh 2010 [57] | Case-crossover |  | multiple monitors, assumed <25% missing data | specification of weather variables not described |  |  |  |
| Huang 2016 [58] | Case-crossover |  | multiple monitors, assumed <25% missing data | unidirectional control selection |  |  |  |
| Kojima 2014 [59] | Case-crossover |  | single monitor, <25% missing |  |  |  |  |
| Li 2019 [60] | Case-crossover |  | multiple monitors, assumed <25% missing | no adjustment for holidays, influenza |  |  |  |
| Liu 2017 [61] | Case-crossover |  | multiple monitors, assumed <25% missing data | specification of weather variables not described |  |  |  |
| Milojevic 2014 [44] | Case-crossover |  |  | no adjustment for holidays, influenza |  |  |  |
| Nuvolone 2011 [45] | Case-crossover |  |  |  |  |  |  |
| Panasevich 2013 [46] | Case-crossover |  | single monitor, <25% missing | temperature specification not described |  |  | author employed by drug company |
| Peel 2007 [32] | Case-crossover |  |  | no adjustment for holidays, influenza |  |  | partially supported by Electric Power Research Institute |
| Peters 2001 [33] | Case-crossover |  | single monitor, < 25% missing data |  |  |  |  |
| Peters 2005 [47] | Case-crossover |  | single monitor, < 25% missing data | no covariates- little evidence of confounding |  |  |  |
| Rich 2010 [34] | Case-crossover |  | assumed<25% missing | no adjustment for holidays, influenza |  |  |  |
| Ruidavets 2005 [48] | Case-crossover |  |  | linear specification of weather without justification, no adjustment for holidays |  |  |  |
| Sahlen 2019 [49] | Case-crossover |  | single monitor, <25% missing | no adjustment for holidays, influenza |  |  |  |
| Tsai 2012 [62] | Case-crossover |  | multiple monitors, assumed <25% missing | no adjustment for holidays, influenza |  |  |  |
| Turin 2012 [63] | Case-crossover |  | single monitor located 20 km away with low correlation (0.5) with local measurements | linear specification of weather without justification, no adjustment for holidays, influenza |  |  |  |
| Vencloviene 2011 [50] | Case-crossover |  | single monitor, assumed <25% missing data | specification of weather variables not described |  |  |  |
| Wang 2015 [26] | Case-crossover |  | multiple monitors, assumed <25% missing | specification of weather variables not described |  |  |  |
| Wang 2015 [27] | Case-crossover |  | multiple monitors, assumed <25% missing data | specification of weather variables not described |  |  |  |
| Weichenthal 2016 [28] | Case-crossover |  | multiple monitors, assumed <25% missing data | no adjustment for holidays, influenza |  |  |  |
| Weichenthal 2016 [29] | Case-crossover |  | multiple monitors, assumed <25% missing data | no adjustment for holidays, influenza |  |  |  |
| Wichmann 2012 [51] | Case-crossover |  | single monitor, <25% missing |  |  |  |  |
| Wichmann 2013 [52] | Case-crossover |  | single monitor, <25% missing | no adjustment for influenza |  |  |  |
| Zanobetti 2006 [35] | Case-crossover |  | multiple monitors, assumed <25% missing | no adjustment for holidays, influenza |  |  |  |
| Anderson 2001 [75] | Time-series |  |  | non-parametric smoothers |  |  |  |
| Atkinson 1999 [76] | Time-series |  | multiple monitors, assumed <25% missing | no adjustment for holidays |  |  |  |
| Baneras 2018 [77] | Time-series |  |  | weather terms not retained in final model |  |  |  |
| Bell 2008 [90] | Time-series |  | multiple monitors, assumed <25% missing | no adjustment for holidays, influenza |  |  |  |
| Burnett 1999 [64] | Time-series |  | multiple monitors, assumed <25% missing | non-parametric smoothers of time, weather, no adjustment for holidays, influenza |  |  |  |
| Caussin 2015 [78] | Time-series |  |  |  |  |  |  |
| Cendon 2006 [91] | Time-series |  |  | non-parametric smoothers, no adjustment for holidays, influenza |  |  |  |
| Chen 2019 [92] | Time-series |  |  | no adjustment for influenza |  |  |  |
| Collart 2018 [79] | Time-series |  |  | no adjustment for holidays, influenza |  |  |  |
| Eilstein 2001 [80] | Time-series |  |  | non-parametric smoothers |  |  |  |
| Ghaffari 2017 [93] | Time-series |  | multiple monitors, assumed <25% missing | no adjustment for day of week, holidays, influenza, temperature specification not described |  |  |  |
| Goggins 2013 [94] | Time-series |  | number of monitors, % missing not reported | no adjustment for holidays, influenza (2 of 3 cities), time specification not described |  |  |  |
| Halonen 2009 [81] | Tim-series |  | multiple monitors, assumed <25% missing | linear specification of weather without justification |  |  |  |
| Hosseinpoor 2005 [95] | Time-series |  | single monitor, <25% missing | specification of humidity, temperature, time not described, no adjustment for influenza |  |  |  |
| Jalaludin 2006 [96] | Time-series |  | multiple monitors, assumed <25% missing |  |  |  |  |
| Konduracka 2019 [82] | Time-series |  |  | no adjustment for day of week, holidays |  |  |  |
| Krall 2018 [68] | Time-series |  |  | no adjustment for influenza |  |  | partially supported by Electric Power Research Institute |
| Lanki 2006 [83] | Time-series |  | multiple monitors, assumed <25% missing | no adjustment for influenza |  |  |  |
| Larrieu 2007 [84] | Time-series |  | multiple monitors, assumed<25% missing |  |  | up to 44% missing data by centre but unlikely to be associated with variability in NO_2_ |  |
| Le Tertre 2002 [85] | Time-series |  |  |  |  |  |  |
| Lee 2003 [97] | Time-series |  | multiple monitors, assumed <25% missing | non-parametric smoothers of time, weather, no adjustment for holidays, influenza |  |  |  |
| Linn 2000 [69] | Time-series |  |  | no adjustment for influenza |  |  |  |
| Lippmann 2000 [70] | Time-series |  | multiple monitors, assumed <25% missing | non-parametric smoothers of temperature, time |  |  |  |
| Mann 2002 [71] | Time-series |  |  | no adjustment for holidays, influenza |  |  |  |
| Medina 1997 [86] | Time-series |  | multiple monitors, assumed <25% missing data | non-parametric smoothers | Physician visit data not coded according to ICD classification or clinical criteria. |  |  |
| Metzger 2004 [72] | Time-series |  |  | no adjustment for influenza |  |  | partially supported by Electric Power Research Institute |
| Pearce 2018 [73] | Time-series |  |  | no adjustment for holidays, influenza |  |  |  |
| Phosri 2019 [98] | Time-series |  | multiple monitors, assumed < 25% missing | no adjustment for holidays, influenza |  |  |  |
| Poloniecki 1997 [87] | Time-series |  | single monitor, assumed <25% missing | linear specification for weather without justification |  |  |  |
| Ponka 1996 [88] | Time-series |  |  | weather specification not described, no adjustment for holidays |  |  |  |
| Pothirat 2019 [99] | Time-series |  | single monitor | linear specification for weather without justification |  |  |  |
| Qiu 2013 [100] | Time-series |  | multiple monitors, assumed <25% missing | non-parametric smoothers |  |  |  |
| Sarnat 2015 [74] | Time-series |  |  | no adjustment for influenza |  |  | partially supported by Electric Power Research Institute |
| Simpson 2005 [101] | Time-series |  |  |  |  |  |  |
| Soleimani 2019 [102] | Time-series |  | number of monitors not specified | specification of weather variables not described | not coded using ICD or explicit diagnostic criteria |  |  |
| Stieb 2000 [65] | Time-series |  |  | non-parametric smoothers of time, weather, no adjustment for holidays, influenza |  |  |  |
| Stieb 2009 [66] | Time-series |  |  | no adjustment for influenza |  |  |  |
| Szyszkowicz 2007 [67] | Time-series |  |  | no adjustment for holidays, influenza, weather specified as linear without justification |  |  |  |
| Tam 2015 [103] | Time-series |  |  | non-parametric smoothers |  |  |  |
| Thach 2010 [104] | Time-series |  |  |  |  |  |  |
| von Klot 2005 [89] | Time-series |  |  | no adjustment for influenza |  |  |  |
| Wong 1999 [105] | Time-series |  | multiple monitors, assumed <25% missing | linear specification for weather without justification, no adjustment for influenza |  |  |  |
| Wong 2002 [106] | Time-series |  |  | non-parametric smoothers for humidty, temperature, time |  |  |  |
| Xie 2014 [107] | Time-series |  | single monitor, assume <25% missing data | no adjustment for influenza |  |  |  |
| Yamaji 2017 [108] | Time-series |  |  | no adjustment for influenza |  |  |  |
| Ye 2001 [109] | Time-series |  |  | no adjustment for holidays |  |  |  |
| Yu 2013 [110] | Time-series |  | multiple monitors, assumed <25% missing | non-parametric smoothers |  |  |  |
| Yu 2018 [111] | Time-series |  |  | no adjustment for holidays, influenza |  |  |  |
