# Supplementary figures and images for: Systematic review and meta-analysis of case-crossover and time-series studies of short term outdoor nitrogen dioxide exposure and ischemic heart disease morbidity
Source: Environ Health. 2020 May 1;19:47. doi: 10.1186/s12940-020-00601-1 (PMC7195719; doi:10.1186/s12940-020-00601-1)

## Canada

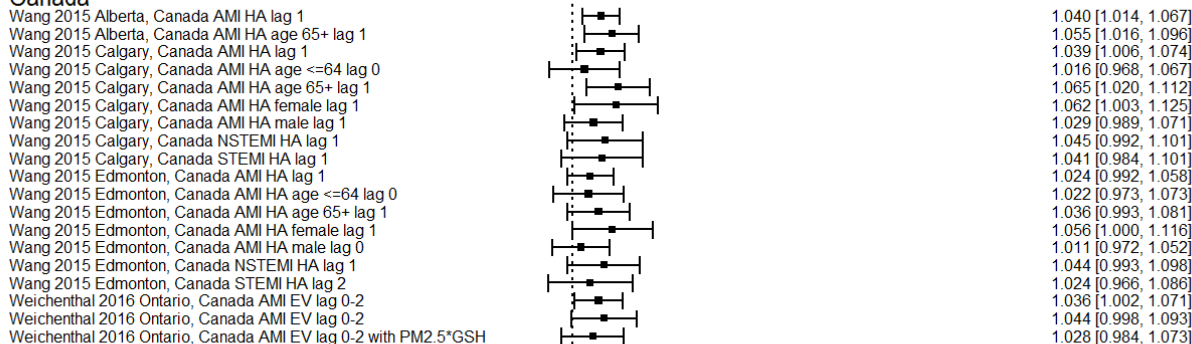

## United States

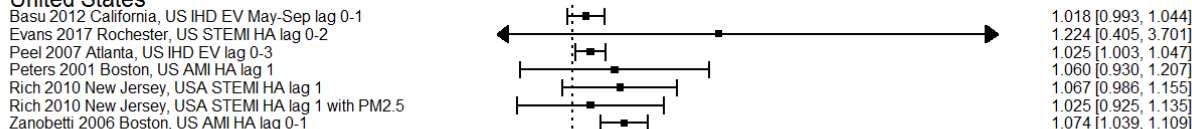

## Europe

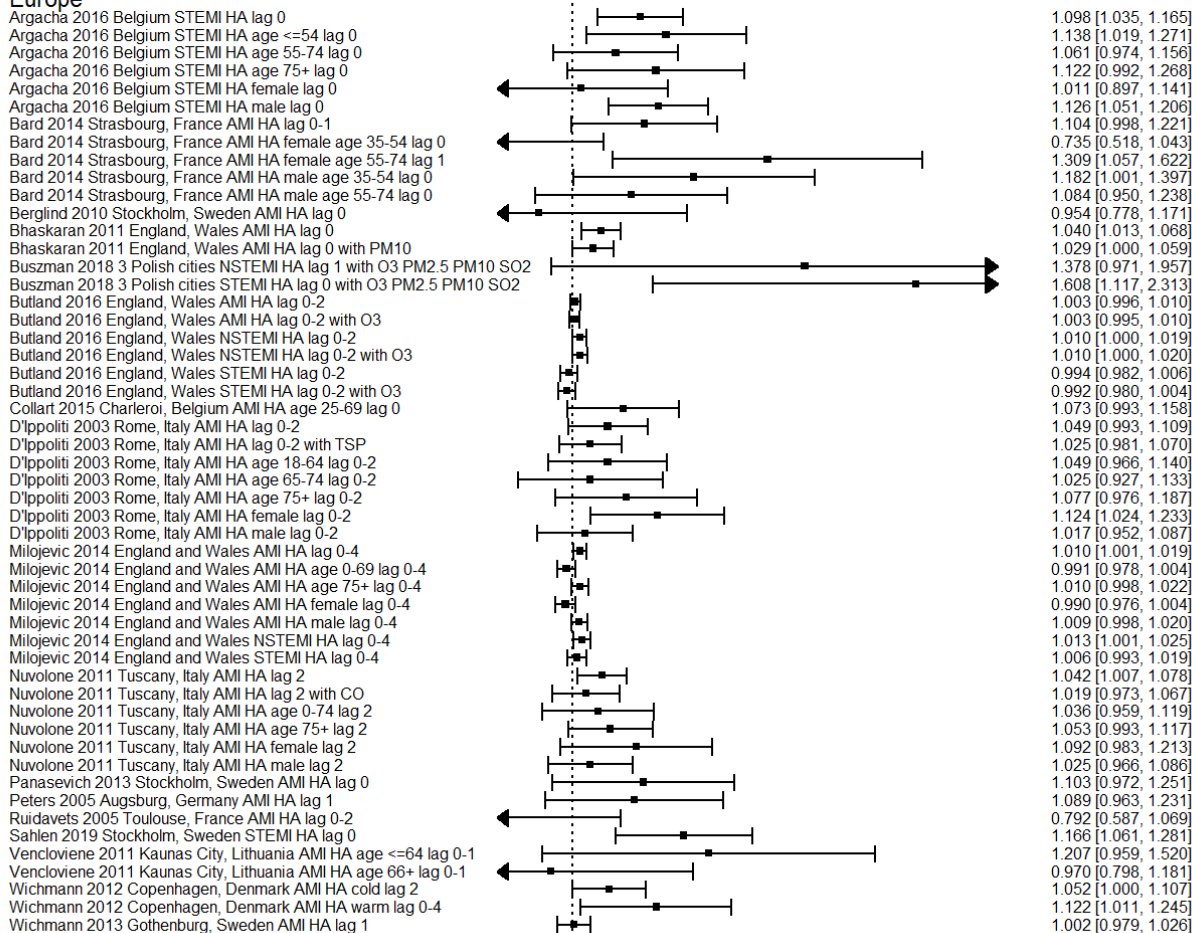

0.9 1 1.2 1.4 1.6 1.8

Odds Ratio, 95% Confidence Interval per 10 ppb NO<sub>2</sub>

Supplement: Supplementary file 4 — Additional file 4. Forest plot of case-crossover studies from Europe and North America (AMI, acute myocardial infarction, NSTEMI, non ST-elevation MI, STEMI, ST-elevation MI, EV, emergency visit, HA, hospital admission, T, temperature, Ox, total oxidants, GSH, glutathione related oxidative potential). [file 12940_2020_601_MOESM4_ESM.pdf]
