# Supplementary material for: Systematic review and meta-analysis of case-crossover and time-series studies of short term outdoor nitrogen dioxide exposure and ischemic heart disease morbidity
Source: Environ Health. 2020 May 1;19:47. doi: 10.1186/s12940-020-00601-1 (PMC7195719; doi:10.1186/s12940-020-00601-1)
Supplement: Supplementary file 5 — Additional file 5. Forest plot of case-crossover studies outside Europe and North America (AMI, acute myocardial infarction, STEMI, ST-elevation MI, EV, emergency visit, HA, hospital admission, T, temperature). [file 12940_2020_601_MOESM5_ESM.pdf]

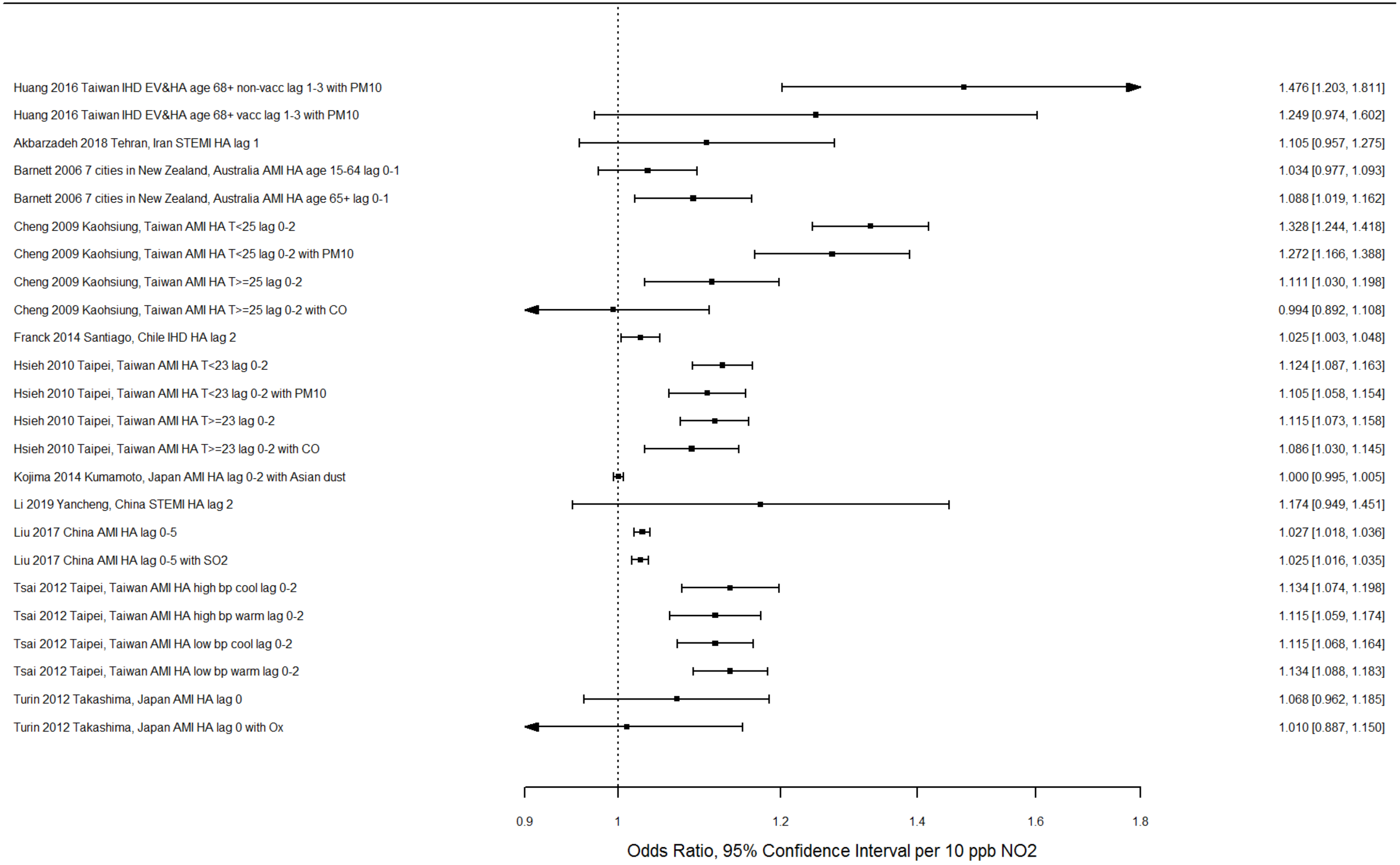

Additional File 5 – Forest plot of case-crossover studies outside Europe and North America (AMI, acute myocardial infarction, STEMI, ST-elevation MI, EV, emergency visit, HA, hospital admission, T, temperature)
