# Supplementary material for: Systematic review and meta-analysis of case-crossover and time-series studies of short term outdoor nitrogen dioxide exposure and ischemic heart disease morbidity
Source: Environ Health. 2020 May 1;19:47. doi: 10.1186/s12940-020-00601-1 (PMC7195719; doi:10.1186/s12940-020-00601-1)
Supplement: Supplementary file 7 — Additional file 7. Forest plot of time-series studies from outside Europe and North America (AMI, acute myocardial infarction, AP, angina pectoris, IHD, ischemic heart disease, STEMI, ST-elevation MI, EV, emergency visit, HA, hospital admission, lag reported in days). [file 12940_2020_601_MOESM7_ESM.pdf]

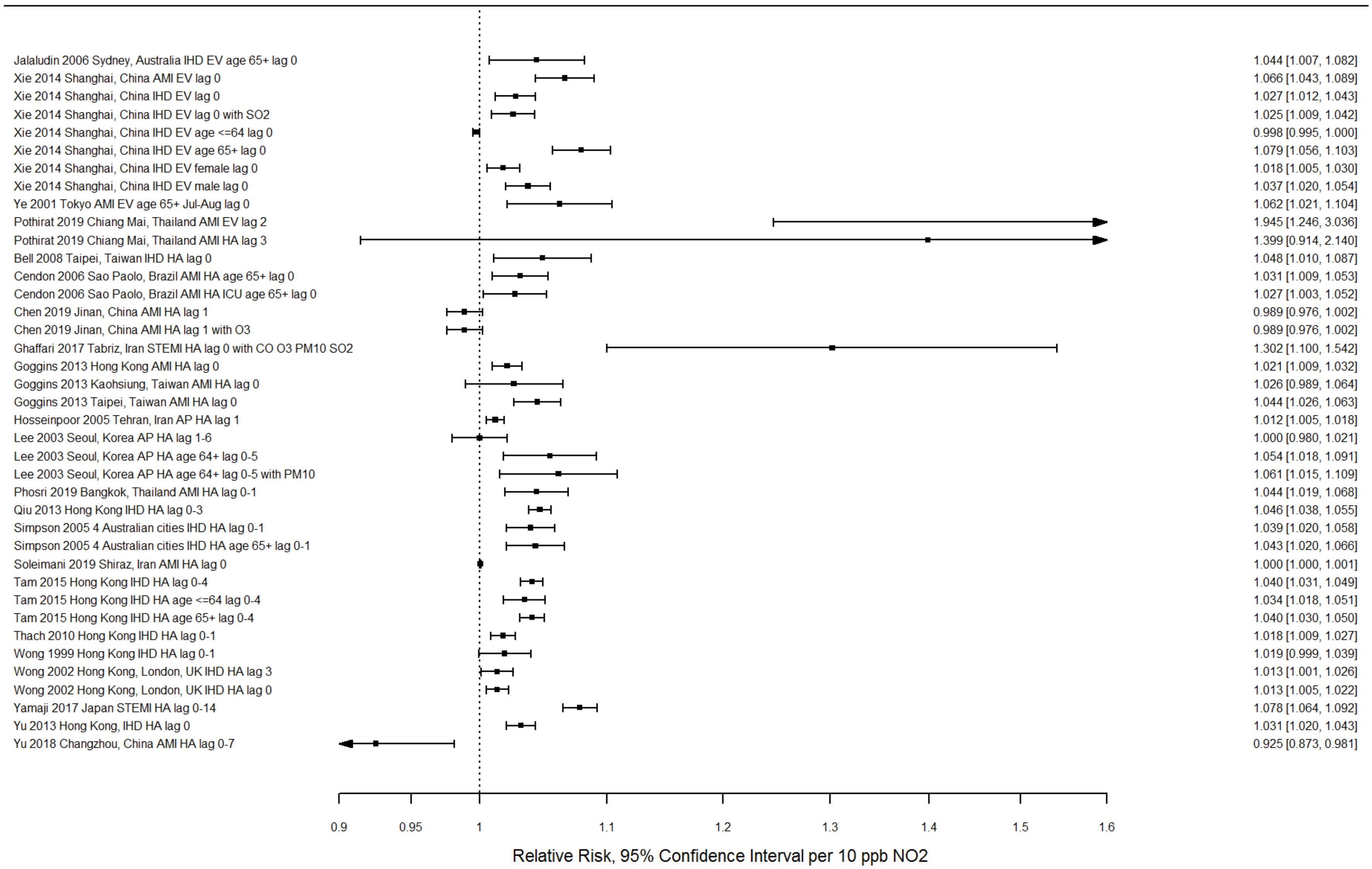

Additional File 7 - Forest plot of time-series studies from outside Europe and North America (AMI, acute myocardial infarction, AP, angina pectoris, IHD, ischemic heart disease, STEMI, ST-elevation MI, EV, emergency visit, HA, hospital admission, lag reported in days)
