# Supplementary material for: Systematic review and meta-analysis of case-crossover and time-series studies of short term outdoor nitrogen dioxide exposure and ischemic heart disease morbidity
Source: Environ Health. 2020 May 1;19:47. doi: 10.1186/s12940-020-00601-1 (PMC7195719; doi:10.1186/s12940-020-00601-1)
Supplement: Supplementary file 8 — Additional file 8. Sensitivity analyses by estimator. [file 12940_2020_601_MOESM8_ESM.docx]

**Additional File 8 - Sensitivity analyses by estimator**

**Case-crossover**

**Random-Effects Model (k = 34; tau^2 estimator: REML)**

tau^2 (estimated amount of total heterogeneity): 0.0024 (SE = 0.0009)

tau (square root of estimated tau^2 value): 0.0495

I^2 (total heterogeneity / total variability): 91.05%

H^2 (total variability / sampling variability): 11.18

Test for Heterogeneity:

Q(df = 33) = 212.9101, p-val < .0001

Model Results:

estimate se zval pval ci.lb ci.ub

0.0717 0.0107 6.7148 <.0001 0.0508 0.0927 ***

**Random-Effects Model (k = 34; tau^2 estimator: DL)**

tau^2 (estimated amount of total heterogeneity): 0.0013 (SE = 0.0010)

tau (square root of estimated tau^2 value): 0.0362

I^2 (total heterogeneity / total variability): 84.50%

H^2 (total variability / sampling variability): 6.45

Test for Heterogeneity:

Q(df = 33) = 212.9101, p-val < .0001

Model Results:

estimate se zval pval ci.lb ci.ub

0.0684 0.0085 8.0216 <.0001 0.0517 0.0851 ***

**Random-Effects Model (k = 34; tau^2 estimator: EB)**

tau^2 (estimated amount of total heterogeneity): 0.0023 (SE = 0.0009)

tau (square root of estimated tau^2 value): 0.0484

I^2 (total heterogeneity / total variability): 90.71%

H^2 (total variability / sampling variability): 10.76

Test for Heterogeneity:

Q(df = 33) = 212.9101, p-val < .0001

Model Results:

estimate se zval pval ci.lb ci.ub

0.0716 0.0105 6.8040 <.0001 0.0509 0.0922 ***

**Time-series**

**Random-Effects Model (k = 41; tau^2 estimator: REML)**

tau^2 (estimated amount of total heterogeneity): 0.0003 (SE = 0.0001)

tau (square root of estimated tau^2 value): 0.0175

I^2 (total heterogeneity / total variability): 95.41%

H^2 (total variability / sampling variability): 21.78

Test for Heterogeneity:

Q(df = 40) = 589.0687, p-val < .0001

Model Results:

estimate se zval pval ci.lb ci.ub

0.0221 0.0033 6.7808 <.0001 0.0157 0.0285 ***

**Random-Effects Model (k = 41; tau^2 estimator: DL)**

tau^2 (estimated amount of total heterogeneity): 0.0002 (SE = 0.0002)

tau (square root of estimated tau^2 value): 0.0142

I^2 (total heterogeneity / total variability): 93.21%

H^2 (total variability / sampling variability): 14.73

Test for Heterogeneity:

Q(df = 40) = 589.0687, p-val < .0001

Model Results:

estimate se zval pval ci.lb ci.ub

0.0218 0.0028 7.8624 <.0001 0.0164 0.0272 ***

**Random-Effects Model (k = 41; tau^2 estimator: EB)**

tau^2 (estimated amount of total heterogeneity): 0.0006 (SE = 0.0002)

tau (square root of estimated tau^2 value): 0.0246

I^2 (total heterogeneity / total variability): 97.62%

H^2 (total variability / sampling variability): 42.09

Test for Heterogeneity:

Q(df = 40) = 589.0687, p-val < .0001

Model Results:

estimate se zval pval ci.lb ci.ub

0.0226 0.0043 5.2109 <.0001 0.0141 0.0311 ***
