# Supplementary material for: Systematic review and meta-analysis of case-crossover and time-series studies of short term outdoor nitrogen dioxide exposure and ischemic heart disease morbidity
Source: Environ Health. 2020 May 1;19:47. doi: 10.1186/s12940-020-00601-1 (PMC7195719; doi:10.1186/s12940-020-00601-1)
Supplement: Supplementary file 9 — Additional file 9. Leave one out analysis. [file 12940_2020_601_MOESM9_ESM.docx]

Additional File 9 - Leave one out analysis - Case-crossover studies

| Omitted Study | Pooled OR | L95%CI | U95%CI | Q | p(Q) | I^2^ (%) |
| --- | --- | --- | --- | --- | --- | --- |
| Akbarzadeh 2018 Tehran, Iran STEMI HA lag 1 | 1.074 | 1.051 | 1.097 | 211.848 | 0.000 | 91.420 |
| Argacha 2016 Belgium STEMI HA lag 0 | 1.074 | 1.051 | 1.097 | 207.557 | 0.000 | 91.511 |
| Bard 2014 Strasbourg, France AMI HA lag 0-1 | 1.074 | 1.051 | 1.097 | 210.788 | 0.000 | 91.463 |
| Barnett 2006 7 cities in New Zealand, Australia AMI HA age 15-64 lag 0-1 | 1.076 | 1.053 | 1.100 | 212.809 | 0.000 | 91.464 |
| Barnett 2006 7 cities in New Zealand, Australia AMI HA age 65+ lag 0-1 | 1.074 | 1.051 | 1.098 | 209.637 | 0.000 | 91.554 |
| Butland 2016 England, Wales AMI HA lag 0-2 | 1.078 | 1.055 | 1.101 | 148.077 | 0.000 | 85.261 |
| Cheng 2009 Kaohsiung, Taiwan AMI HA T<25 lag 0-2 | 1.063 | 1.046 | 1.081 | 152.283 | 0.000 | 83.517 |
| Cheng 2009 Kaohsiung, Taiwan AMI HA T>=25 lag 0-2 | 1.073 | 1.051 | 1.097 | 208.512 | 0.000 | 91.444 |
| Collart 2015 Charleroi, Belgium AMI HA age 25-69 lag 0 | 1.075 | 1.052 | 1.098 | 211.523 | 0.000 | 91.569 |
| D'Ippoliti 2003 Rome, Italy AMI HA lag 0-2 | 1.076 | 1.052 | 1.099 | 212.157 | 0.000 | 91.555 |
| Evans 2017 Rochester, US STEMI HA lag 0-2 | 1.074 | 1.052 | 1.097 | 212.811 | 0.000 | 91.304 |
| Franck 2014 Santiago, Chile IHD HA lag 2 | 1.077 | 1.054 | 1.100 | 212.907 | 0.000 | 90.825 |
| Li 2019 Yancheng, China STEMI HA lag 2 | 1.074 | 1.051 | 1.096 | 211.328 | 0.000 | 91.311 |
| Liu 2017 China AMI HA lag 0-5 | 1.077 | 1.054 | 1.100 | 212.370 | 0.000 | 87.286 |
| Nuvolone 2011 Tuscany, Italy AMI HA lag 2 | 1.076 | 1.053 | 1.100 | 211.946 | 0.000 | 91.393 |
| Panasevich 2013 Stockholm, Sweden AMI HA lag 0 | 1.074 | 1.051 | 1.097 | 211.593 | 0.000 | 91.440 |
| Peel 2007 Atlanta, US IHD EV lag 0-3 | 1.077 | 1.054 | 1.100 | 212.909 | 0.000 | 90.777 |
| Peters 2005 Augsburg, Germany AMI HA lag 1 | 1.074 | 1.052 | 1.097 | 211.967 | 0.000 | 91.468 |
| Rich 2010 New Jersey, USA STEMI HA lag 1 | 1.075 | 1.052 | 1.098 | 211.866 | 0.000 | 91.567 |
| Ruidavets 2005 Toulouse, France AMI HA lag 0-2 | 1.076 | 1.054 | 1.099 | 210.079 | 0.000 | 91.232 |
| Sahlen 2019 Stockholm, Sweden STEMI HA lag 0 | 1.072 | 1.050 | 1.095 | 205.656 | 0.000 | 91.079 |
| Tsai 2012 Taipei, Taiwan AMI HA high bp cool lag 0-2 | 1.072 | 1.050 | 1.095 | 199.442 | 0.000 | 91.146 |
| Tsai 2012 Taipei, Taiwan AMI HA high bp warm lag 0-2 | 1.073 | 1.050 | 1.096 | 202.393 | 0.000 | 91.360 |
| Tsai 2012 Taipei, Taiwan AMI HA low bp cool lag 0-2 | 1.073 | 1.050 | 1.096 | 197.705 | 0.000 | 91.308 |
| Tsai 2012 Taipei, Taiwan AMI HA low bp warm lag 0-2 | 1.072 | 1.049 | 1.095 | 190.192 | 0.000 | 91.018 |
| Turin 2012 Takashima, Japan AMI HA lag 0 | 1.075 | 1.052 | 1.098 | 212.296 | 0.000 | 91.518 |
| Vencloviene 2011 Kaunas City, Lithuania AMI HA age <=64 lag 0-1 | 1.073 | 1.051 | 1.096 | 210.953 | 0.000 | 91.281 |
| Vencloviene 2011 Kaunas City, Lithuania AMI HA age 66+ lag 0-1 | 1.075 | 1.053 | 1.098 | 212.617 | 0.000 | 91.351 |
| Wang 2015 Alberta, Canada AMI HA lag 1 | 1.076 | 1.053 | 1.100 | 211.492 | 0.000 | 91.189 |
| Weichenthal 2016 Ontario, Canada AMI EV lag 0-2 | 1.076 | 1.053 | 1.099 | 212.232 | 0.000 | 91.502 |
| Wichmann 2012 Copenhagen, Denmark AMI HA cold lag 2 | 1.075 | 1.052 | 1.099 | 211.840 | 0.000 | 91.558 |
| Wichmann 2012 Copenhagen, Denmark AMI HA warm lag 0-4 | 1.073 | 1.051 | 1.097 | 209.957 | 0.000 | 91.394 |
| Wichmann 2013 Gothenburg, Sweden AMI HA lag 1 | 1.078 | 1.055 | 1.101 | 209.326 | 0.000 | 90.360 |
| Zanobetti 2006 Boston, US AMI HA lag 0-1 | 1.075 | 1.051 | 1.098 | 204.727 | 0.000 | 91.486 |

Time-series studies

| Omitted Study | Pooled RR | L95%CI | U95%CI | Q | p(Q) | I^2^ (%) |
| --- | --- | --- | --- | --- | --- | --- |
| Anderson 2001 West Midland, UK IHD HA age 65+ lag 0-1 | 1.023 | 1.016 | 1.029 | 587.865 | 0.000 | 95.627 |
| Baneras 2015 Barcelona STEMI HA lag 2 | 1.023 | 1.016 | 1.030 | 586.087 | 0.000 | 93.398 |
| Bell 2008 Taipei, Taiwan IHD HA lag 0 | 1.022 | 1.015 | 1.029 | 583.072 | 0.000 | 95.504 |
| Burnett 1999 Toronto, Canada IHD HA lag 0-1 | 1.022 | 1.015 | 1.029 | 517.104 | 0.000 | 95.390 |
| Caussin 2015 Paris, France STEMI HA lag 1 | 1.023 | 1.016 | 1.029 | 589.067 | 0.000 | 95.534 |
| Cendon 2006 Sao Paolo, Brazil AMI HA age 65+ lag 0 | 1.022 | 1.015 | 1.029 | 581.794 | 0.000 | 95.613 |
| Cendon 2006 Sao Paolo, Brazil AMI HA ICU age 65+ lag 0 | 1.022 | 1.016 | 1.029 | 584.717 | 0.000 | 95.630 |
| Chen 2019 Jinan, China AMI HA lag 1 | 1.023 | 1.017 | 1.030 | 585.579 | 0.000 | 95.017 |
| Collart 2018 Wallonia, Belgium AMI HA lag 0 | 1.022 | 1.015 | 1.028 | 582.057 | 0.000 | 95.465 |
| Eilstein 2001 Strasbourg, France AMI HA lag 5 | 1.023 | 1.016 | 1.029 | 586.090 | 0.000 | 95.633 |
| Goggins 2013 Kaohsiung, Taiwan AMI HA lag 0 | 1.022 | 1.016 | 1.029 | 587.362 | 0.000 | 95.607 |
| Goggins 2013 Taipei, Taiwan AMI HA lag 0 | 1.022 | 1.015 | 1.028 | 567.713 | 0.000 | 95.437 |
| Halonen 2009 Helsinki, Finland IHD HA age 65+ lag 2 | 1.023 | 1.016 | 1.029 | 588.829 | 0.000 | 95.590 |
| Hosseinpoor 2005 Tehran, Iran AP HA lag 1 | 1.023 | 1.016 | 1.030 | 579.905 | 0.000 | 95.389 |
| Jalaludin 2006 Sydney, Australia IHD EV age 65+ lag 0 | 1.022 | 1.015 | 1.029 | 583.916 | 0.000 | 95.535 |
| Konduracka 2019 Krakow, Poland AMI HA age 70+ lag 0-1 | 1.022 | 1.016 | 1.029 | 583.228 | 0.000 | 95.484 |
| Krall 2018 5 U.S. cities IHD EV lag 0 | 1.022 | 1.015 | 1.029 | 578.883 | 0.000 | 95.528 |
| Lanki 2006 5 European cities AMI HA lag 0 | 1.023 | 1.017 | 1.030 | 587.973 | 0.000 | 95.273 |
| Larrieu 2007 8 French cities IHD HA lag 0-1 | 1.022 | 1.015 | 1.029 | 574.672 | 0.000 | 95.592 |
| Le Tertre 2002 8 European cities IHD HA age <=64 lag 0-1 | 1.023 | 1.016 | 1.030 | 584.415 | 0.000 | 95.262 |
| Le Tertre 2002 8 European cities IHD HA age 65+ lag 0-1 | 1.023 | 1.016 | 1.029 | 544.084 | 0.000 | 95.267 |
| Lee 2003 Seoul, Korea AP HA lag 1-6 | 1.023 | 1.016 | 1.030 | 589.053 | 0.000 | 95.476 |
| Linn 2000 Los Angeles, US AMI HA age 30+ lag 0 | 1.023 | 1.016 | 1.030 | 585.299 | 0.000 | 95.525 |
| Lippmann 2000 Detroit, US IHD HA age 65+ lag 1-2 | 1.023 | 1.016 | 1.029 | 586.653 | 0.000 | 95.638 |
| Mann 2002 California, US AMI HA lag 0 | 1.022 | 1.016 | 1.029 | 574.351 | 0.000 | 95.583 |
| Medina 1997 Paris, France IHD MD lag 2 | 1.022 | 1.016 | 1.029 | 586.028 | 0.000 | 95.502 |
| Metzger 2004 Atlanta, US IHD EV lag 0-3 | 1.022 | 1.016 | 1.029 | 583.843 | 0.000 | 95.630 |
| Pearce 2018 Columbia, US IHD HA lag 0 | 1.022 | 1.015 | 1.028 | 555.381 | 0.000 | 95.316 |
| Phosri 2019 Bangkok, Thailand AMI HA lag 0-1 | 1.022 | 1.015 | 1.028 | 577.138 | 0.000 | 95.491 |
| Poloniecki 1997 London, UK AMI HA lag 1 | 1.023 | 1.016 | 1.030 | 583.491 | 0.000 | 95.327 |
| Pothirat 2019 Chiang Mai, Thailand AMI EV lag 2 | 1.022 | 1.016 | 1.029 | 580.522 | 0.000 | 95.502 |
| Pothirat 2019 Chiang Mai, Thailand AMI HA lag 3 | 1.022 | 1.016 | 1.029 | 586.693 | 0.000 | 95.514 |
| Simpson 2005 4 Australian cities IHD HA lag 0-1 | 1.022 | 1.015 | 1.029 | 573.154 | 0.000 | 95.527 |
| Soleimani 2019 Shiraz, Iran AMI HA lag 0 | 1.023 | 1.017 | 1.030 | 330.105 | 0.000 | 90.150 |
| Stieb 2009 7 Canadian cities IHD EV lag 1 | 1.023 | 1.016 | 1.029 | 585.401 | 0.000 | 95.609 |
| Tam 2015 Hong Kong IHD HA lag 0-4 | 1.022 | 1.015 | 1.028 | 509.056 | 0.000 | 95.344 |
| Thach 2010 Hong Kong IHD HA lag 0-1 | 1.023 | 1.016 | 1.029 | 576.566 | 0.000 | 95.560 |
| Wong 1999 Hong Kong IHD HA lag 0-1 | 1.022 | 1.016 | 1.029 | 586.024 | 0.000 | 95.642 |
| Xie 2014 Shanghai, China IHD EV lag 0 | 1.022 | 1.016 | 1.029 | 578.047 | 0.000 | 95.626 |
| Yamaji 2017 Japan STEMI HA lag 0-14 | 1.020 | 1.015 | 1.026 | 464.860 | 0.000 | 93.278 |
| Yu 2018 Changzhou, China AMI HA lag 0-7 | 1.023 | 1.017 | 1.030 | 582.057 | 0.000 | 95.167 |
