# Supplementary material for: Systematic review and meta-analysis of case-crossover and time-series studies of short term outdoor nitrogen dioxide exposure and ischemic heart disease morbidity
Source: Environ Health. 2020 May 1;19:47. doi: 10.1186/s12940-020-00601-1 (PMC7195719; doi:10.1186/s12940-020-00601-1)

Additional File 10 - Funnel plot of log(Relative Risk) vs. standard error for time-series studies from Figure 4. Filled circles represent observed values, open circles represent missing studies identified with trim and fill, and the vertical line represents the log of the pooled relative risk. In the absence of publication bias, points should be symmetrically distributed around the vertical line, with smaller studies (larger standard errors on vertical axis) more widely scattered. Filling the plot with points mirroring observed values corrects for apparently missing smaller and/or negative studies which may have been suppressed due to publication bias.


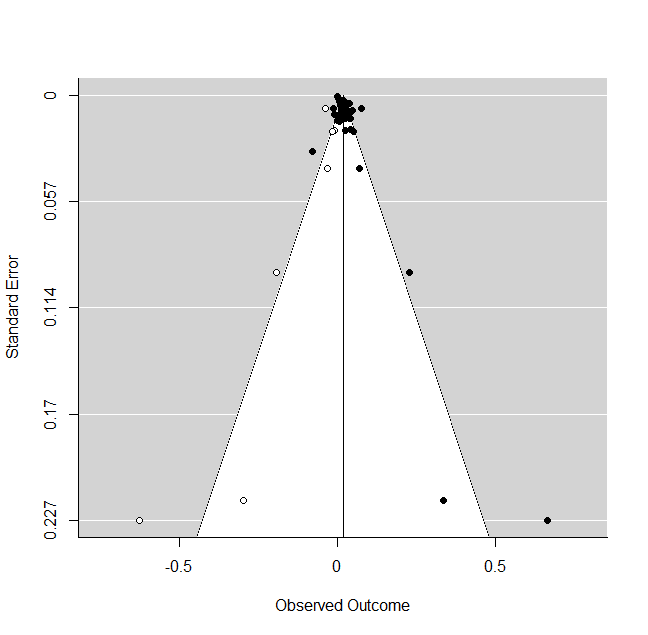

Supplement: Supplementary file 10 — Additional file 10. Funnel plot of log(Relative Risk) vs. standard error for time-series studies from Fig. 4. Filled circles represent observed values, open circles represent missing studies identified with trim and fill, and the vertical line represents the log of the pooled relative risk. In the absence of publication bias, points should be symmetrically distributed around the vertical line, with smaller studies (larger standard errors on vertical axis) more widely scattered. Filling the plot with points mirroring observed values corrects for apparently missing smaller and/or negative studies which may have been suppressed due to publication bias. [file 12940_2020_601_MOESM10_ESM.docx]
