# Supplementary material for: Systematic review and meta-analysis of case-crossover and time-series studies of short term outdoor nitrogen dioxide exposure and ischemic heart disease morbidity
Source: Environ Health. 2020 May 1;19:47. doi: 10.1186/s12940-020-00601-1 (PMC7195719; doi:10.1186/s12940-020-00601-1)
Supplement: Supplementary file 11 — Additional file 11. Navigation Guide Criteria for Overall Quality and Strength of Evidencea. [file 12940_2020_601_MOESM11_ESM.docx]

Additional File 11 – Navigation Guide Criteria for Overall Quality and Strength of Evidence^a^

| Downgrading Factors | Summary of criteria for downgrading |
| --- | --- |
| Risk of bias | Study limitations – a substantial risk of bias across body of evidence |
| Indirectness | Evidence was not directly comparable to the question of interest (i.e., population, exposure, comparator, outcome) |
| Inconsistency | Widely different estimates of effect in similar populations (heterogeneity or variability in results) |
| Imprecision | Studies had few participants and few events (wide confidence intervals as judged by reviewers) |
| Publication Bias | Studies missing from body of evidence, resulting in an over or underestimate of true effects from exposure |
| Upgrading Factors | Summary of criteria for upgrading |
| Large magnitude of effect | Upgraded if modeling suggested confounding alone unlikely to explain associations with large effect estimate as judged by reviewers |
| Dose response | Upgraded if consistent relationship between dose and response in one or multiple studies, and/or dose response across studies |
| Confounding minimizes effect | Upgraded if consideration of all plausible residual confounders or biases would underestimate the effect or suggest a spurious effect when results show no effect |
| ^a^Adaped from: Lam J, Sutton P, McPartland J, Davidson L, Daniels N, Sen S, et al. Applying the Navigation Guide Systematic Review Methodology Case Study #5 Association between Developmental Exposures to PBDEs and Human Neurodevelopment: A Systematic Review of the Evidence Protocol April 2015 [Internet]. [cited 2019 Aug 18]. Available from: <http://www.crd.york.ac.uk/PROSPEROFILES/17890_PROTOCOL_20150322.pdf> | |
