# Supplementary material for: Systematic review and meta-analysis of case-crossover and time-series studies of short term outdoor nitrogen dioxide exposure and ischemic heart disease morbidity
Source: Environ Health. 2020 May 1;19:47. doi: 10.1186/s12940-020-00601-1 (PMC7195719; doi:10.1186/s12940-020-00601-1)
Supplement: Supplementary file 12 — Additional file 12. USEPA/Health Canada Criteria for Evaluating Likelihood of Causal Relationshipa. [file 12940_2020_601_MOESM12_ESM.docx]

Additional File 12 – USEPA/Health Canada Criteria for Evaluating Likelihood of Causal Relationship^a^

| Causal relationship | Evidence is sufficient to conclude that there is a causal relationship with relevant pollutant exposures (i.e., doses or exposures generally within one to two orders of magnitude of current levels). That is, the pollutant has been shown to result in health effects in studies in which chance, bias, and confounding could be ruled out with reasonable confidence. For example: a) controlled human exposure studies that demonstrate consistent effects; or b) observational studies that cannot be explained by plausible alternatives or are supported by other lines of evidence (e.g., animal studies or mode of action information). Evidence includes multiple high-quality studies. |
| --- | --- |
| Likely to be a causal relationship | Evidence is sufficient to conclude that a causal relationship is likely to exist with relevant pollutant exposures, but important uncertainties remain. That is, the pollutant has been shown to result in health effects in studies in which chance and bias can be ruled out with reasonable confidence but potential issues remain. For example: a) observational studies show an association, but co-pollutant exposures are difficult to address and/or other lines of evidence (controlled human exposure, animal, or mode of action information) are limited or inconsistent; or b) animal toxicological evidence from multiple studies from different laboratories that demonstrate effects, but limited or no human data are available. Evidence generally includes multiple high-quality studies. |
| Suggestive of a causal relationship | Evidence is suggestive of a causal relationship with relevant pollutant exposures, but is limited. For example: a) at least one high-quality epidemiologic study shows an association with a given health outcome but the results of other studies are inconsistent; or b) a well-conducted toxicological study, such as those conducted in the National Toxicology Program (NTP), shows effects in animal species. |
| Inadequate to infer a causal relationship | Evidence is inadequate to determine that a causal relationship exists with relevant pollutant exposures. The available studies are of insufficient quantity, quality, consistency, or statistical power to permit a conclusion regarding the presence or absence of an effect. |
| Not likely to be a causal relationship | Evidence is suggestive of no causal relationship with relevant pollutant exposures. Several adequate studies, covering the full range of levels of exposure that human beings are known to encounter and considering at-risk populations, are mutually consistent in not showing an effect at any level of exposure. |

^a^Health Canada. Human Health Risk Assessment for Ambient Nitrogen Dioxide. Ottawa, Canada: Health Canada; 2016 (Modified from U.S. Environmental Protection Agency. 2013. Integrated science assessment for ozone and

related photochemical oxidants. (EPA/600/R-10/076F). Research Triangle Park, NC: US Environmental Protection

Agency, National Center for Environmental Assessment. <http://cfpub.epa.gov/ncea/isa/recordisplay.cfm?deid=247492>).
